# A novel MYC-ZNF706-SLC7A11 regulatory circuit contributes to cancer progression and redox balance in human hepatocellular carcinoma

**DOI:** 10.1038/s41418-024-01324-3

**Published:** 2024-06-11

**Authors:** Jie Chu, Jun Jiang, Xin Fan, Jun Liu, Ke Gao, Yu Jiang, Mengxuan Li, Wenjin Xi, Lu Zhang, Ka Bian, Angang Yang, Rui Zhang

**Affiliations:** 1 State Key Laboratory of Holistic Integrative Management of Gastrointestinal Cancers, Department of Biochemistry and Molecular Biology, Fourth Military Medical University, Xi’an, Shaanxi 710032 China; 2 Department of Health Service, Base of Health Service, Fourth Military Medical University, Xi’an, Shaanxi 710032 China; 3 Department of Otolaryngology Head and Neck Surgery, Tangdu Hospital, Fourth Military Medical University, Xi’an, Shaanxi 710038 China; 4 Department of Urology, Xi’an People’s Hospital (Xi’an Fourth Hospital), School of Life Sciences and Medicine, Northwest University, Xi’an, Shaanxi 710199 China; 5 State Key Laboratory of Holistic Integrative Management of Gastrointestinal Cancers, Department of Immunology, Fourth Military Medical University, Xi’an, Shaanxi 710032 China

**Keywords:** Oncogenes, Diagnostic markers, Tumour biomarkers

## Abstract

The oncogenic potential of chromosome 8q22 copy number gain in liver cancer remains to be depicted. Here, we report that ZNF706, encoded by a gene mapped to chromosome 8q22, is a C2H2-type zinc finger protein. However, the biological function and mechanism of ZNF706 have been poorly investigated. Clinically, ZNF706 expression was elevated in hepatocellular carcinoma (HCC), and high ZNF706 expression was associated with unfavorable survival in HCC patients. Functional experiments revealed that ZNF706 knockdown inhibited HCC progression both in vitro and in vivo. RNA sequencing (RNA-seq) and chromatin immunoprecipitation-based deep sequencing (ChIP-seq) revealed that mechanistically, ZNF706 is a crucial ferroptosis regulator and that SLC7A11 is a critical target of ZNF706. In addition, ZNF706 knockdown inhibited SLC7A11 expression, increased lipid peroxidation, and promoted ferroptosis. Further analysis revealed that ZNF706 is a novel direct target transcriptionally activated by MYC in HCC cells. Importantly, MYC depletion reduced SLC7A11-mediated redox homeostasis, and this effect was reversed by ZNF706 reexpression. Collectively, our data demonstrate that ZNF706 is a potential oncogene in liver cancer and functions as a ferroptosis regulator by modulating SLC7A11 expression, constituting a potential therapeutic target for HCC.

## Introduction

Hepatocellular carcinoma (HCC) is the most prevalent histological subtype of primary liver cancer and is the third most common cause of cancer mortality worldwide [1–3]. Despite progress in cancer therapy, the prognosis of HCC patients remains exceedingly poor, with a 5-year survival rate of less than 15% owing to the limited availability of clinical therapeutic strategies and the high rate of tumor recurrence [4, 5]. Accumulating evidence indicates that HCC is one of the most rapidly progressing solid tumors, and understanding the mechanism that drives cancer progression is essential for developing more effective therapeutic interventions and improving patient survival.

Genomic abnormalities, which include copy number structural and numerical variations, are critical pathogenic factors of tumors [6, 7]. Copy number gain of chromosome 8q, involving the 8q22 and 8q24 regions, is common in HCC, and high-level amplification is thought to be associated with the rapid progression of HCC and poor clinical outcomes [8, 9]. The MYC gene is accepted as the oncogenic driver of 8q24 gain, however, the oncogenic role of 8q22 gain in liver carcinogenesis is continuing to be elaborated.

Metabolic reprogramming commonly results in oncogene-directed alterations in cancer cells, in which the levels of special nutrients such as glutamine and cystine are altered to maintain cancer cell survival and the intracellular redox balance, eventually facilitating tumor progression [10–12]. Interestingly, cysteine is supposed to be an important amino acid that participates in protein synthesis and the maintenance of redox homeostasis [13]. Cysteine can function as a cellular antioxidant and is the rate-limiting precursor for glutathione (GSH) synthesis [14]. Cancer cells usually acquire cysteine via the uptake of extracellular cystine, with solute carrier family 7 member 11 (SLC7A11), also known as xCT, serving as an important transporter of cystine [15]. When extracellular cystine is imported into the cell via SLC7A11, it is converted into cysteine in an NADPH-dependent reduction reaction. Subsequently, cysteine is used for the production of GSH, which is essential for intracellular antioxidant activity [16]. Therefore, cancer cells, which are often subjected to high levels of oxidative stress, may depend on SLC7A11 to satisfy their growth requirements via cystine import.

Zinc Finger domains account for 5% of all human proteins and interact with various substrates, such as lipids, DNA, RNA, and post-translational modifications [17]. In addition, zinc finger proteins (ZNFs) are involved in transcriptional regulation, signal transduction, and other cellular processes [18]. Zinc finger protein 706 (ZNF706), encoded by a gene located at chromosome 8q22.3, is a member of the C2H2-type zinc finger transcription factor family, the most abundant transcription factor family encoded by the human genome. And according to the structural analysis of ZNF706, it was definitely predicted to physically bind to DNA through the C-terminal zinc finger domain [19]. However, what’s the specific binding motif of ZNF706 on DNA sequences has not been identified. In previous studies, it was reported that the expression of ZNF706 is upregulated in gastric cancer and laryngeal cancer [20, 21]. And ZNF706 may be a biomarker for the early diagnosis of rheumatoid arthritis [22]. However, no study has investigated the functions and mechanisms of ZNF706 in HCC development.

In this study, we found that ZNF706 is frequently upregulated in HCC tissues and cell lines. Using gain and loss of functional experiments, we identified the oncogenic role of ZNF706 in HCC cells in vitro and in vivo. In the mechanistic study, by performing chromatin immunoprecipitation-based deep sequencing (ChIP-seq) and bulk RNA sequencing (RNA-seq), we globally mapped the binding sites of ZNF706 on the genomic DNA of HCC cells and the regulatory patterns of ZNF706 on the downstream genes. Importantly, we identified that ZNF706 is a novel regulator of ferroptosis sensitivity by regulating SLC7A11. In addition, we identified that MYC is an upstream transcriptional factor that stimulates the ZNF706 expression. Collectively, our study demonstrated that a novel MYC-ZNF706-SLC7A11 regulatory circuit promotes advanced progression and regulates redox hemostasis in human hepatocellular carcinoma and especially emphasized the importance of ZNF706 involves etiopathogenesis of HCC progression and its valuable significance as a biomarker and potential therapeutic target for the HCC treatment.

## Results

### High expression of ZNF706 is associated with poor survival in HCC patients

**Fig. 1:**
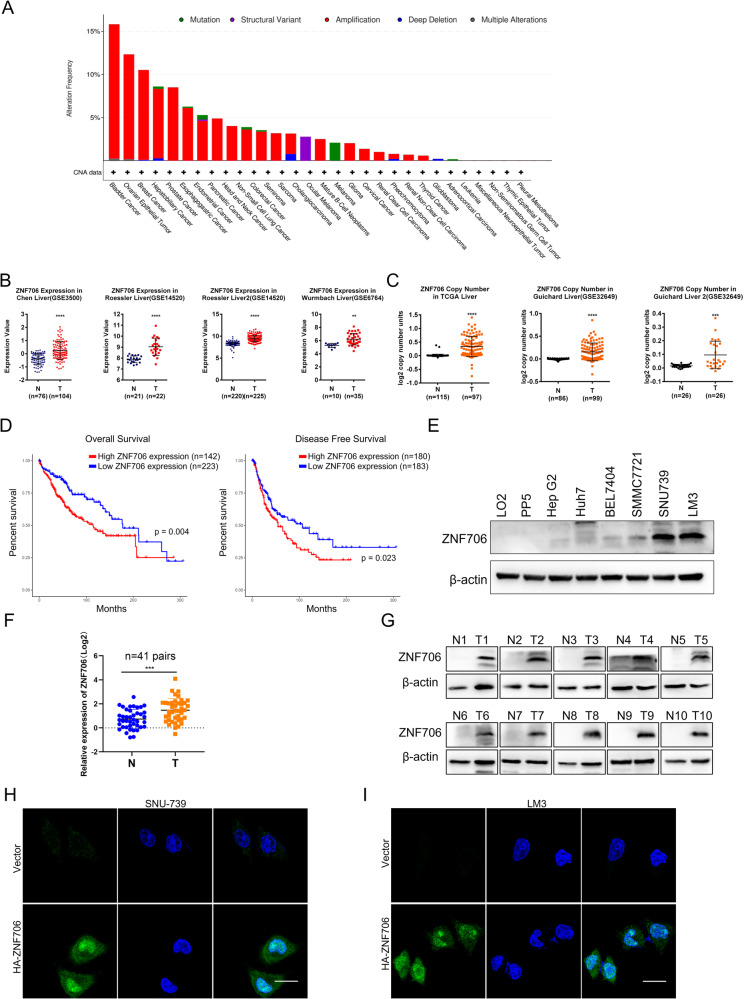
ZNF706 is upregulated in human HCC and predicts poor prognosis of HCC patients. **A** Analysis of genetic alterations of ZNF706 in a variety of cancers from the cBioPortal database. **B** Relative expression of ZNF706 in normal liver and HCC tissues from three GEO datasets GSE3500, GSE14520, and GSE6764. **C** The copy number value of ZNF706 in liver cancer was obtained from the TCGA database and the dataset of GSE32649. **D** High expression of ZNF706 was associated with poor prognosis in liver cancer from the TCGA database. **E** Analysis of the protein levels of ZNF706 in HCC cells. **F**, **G** The protein and mRNA levels of ZNF706 in HCC specimens compared with matched adjacent normal tissues. **H**, **I** The distribution of ZNF706 was measured by immunofluorescent imaging in SNU-739 and LM3 cells. Representative images were shown. Scale bars: 50 μm. All data were shown as mean ± SD, and statistical significance was analyzed by Student *t*-test. **P < 0.01, ***P < 0.001.

By analysis of a publicly available database in the cBioPortal for Cancer Genomics (http://www.cbioportal.org/), we found that ZNF706 exhibited various genetic abnormalities in multiple malignancies, including bladder cancer, ovarian cancer, liver cancer, and prostate cancer. Notably, ZNF706 amplification occurred in approximately 8% of the liver cancer cases with genetic abnormalities (Fig. 1A). Moreover, analysis of mRNA expression data obtained from the HCC datasets GSE3500, GSE14520, and GSE6764 in the Gene Expression Omnibus (GEO) database demonstrated that ZNF706 was obviously overexpressed in liver cancer (Fig. 1B). Moreover, the copy number of ZNF706 was analyzed in The Cancer Genome Atlas (TCGA) and the GSE32649 dataset was found to be significantly increased in liver cancer (Fig. 1C). Further analysis of the TCGA database showed that patients with high ZNF706 expression had a lower survival rate (Fig. 1D). In addition, the ZNF706 level was measured in a panel of HCC cell lines, and the data showed that ZNF706 expression was markedly higher in multiple HCC cell lines than in normal human liver cell lines (Fig. 1E). Furthermore, we analyzed the mRNA expression of ZNF706 in 41 pairs HCC tissues and adjacent normal tissues and found that ZNF706 expression was noticeably elevated in the HCC specimens compared with the paired adjacent normal tissues (Fig. 1F). We then examined the ZNF706 protein level in 10 patients and determined that all HCC specimens exhibited markedly increased ZNF706 expression compared with the paired normal tissues (Fig. 1G). To further confirm the location of ZNF706, SNU-739, and LM3 cells were transfected with a plasmid expressing HA-tagged ZNF706 (HA-ZNF706), and immunofluorescent imaging revealed that ZNF706 was localized primarily in the nucleus (Fig. 1H, I). In addition, we generated an EGFP-ZNF706 fusion (EGFP-ZNF706) expression plasmid, and similar results were obtained (Supplemental Fig. 1A, B). Interestingly, similar results were also obtained in nuclear–cytoplasmic fractionation experiments (Supplemental Fig. 1C). Collectively, these observations suggest that ZNF706 is overexpressed in liver cancer and is associated with poor survival in HCC patients.

### Knockdown of ZNF706 inhibits malignant behaviors of HCC cells in vitro and in vivo

**Fig. 2:**
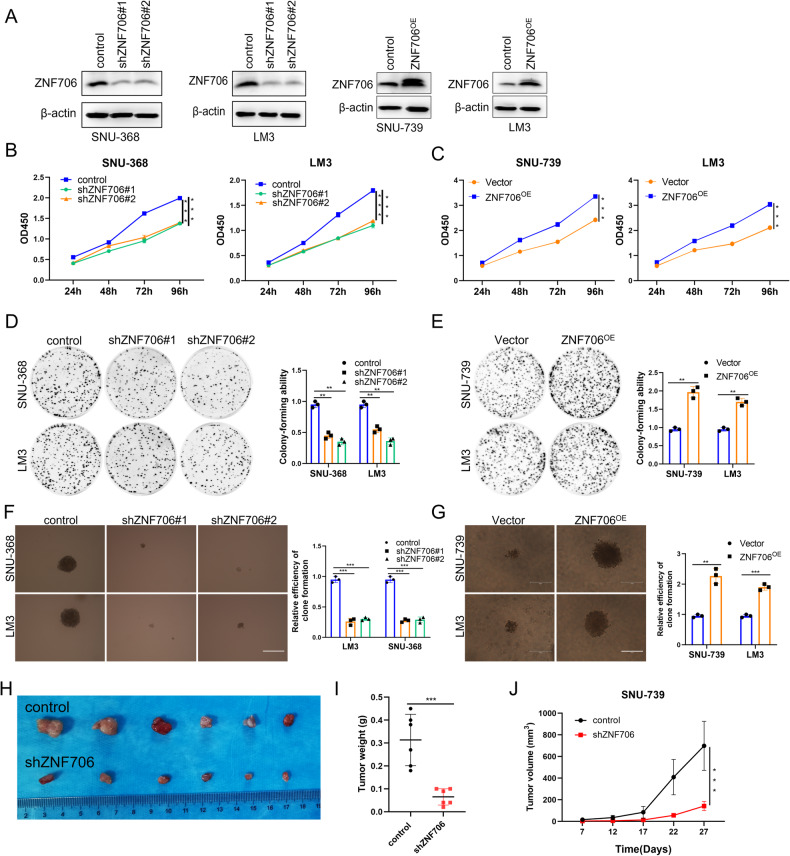
Knockdown of ZNF706 inhibits HCC cells malignant behaviors in vitro and in vivo. **A** Levels of ZNF706 were measured by Western blot assay after SNU-368, LM3 cells with stable depletion of ZNF706 and SNU-739, LM3 cells with ZNF706 Overexpression. **B**, **C** Cell viabilities were measured by Cell counting-8 kit (CCK-8) assay with HCC cells stable depletion and overexpression of ZNF706. **D**, **E** HCC cells with stable ZNF706 knockdown and ZNF706 overexpression were cultured for 10 days to assess the cell proliferation abilities by plate clone assay. **F**, **G** The proliferative abilities of stably depleted or overexpressed ZNF706 HCC cells were examined by soft agar assay. Cells were maintained 14 days before photographed and counted. Scale bars: 150 μm. **H** Xenograft tumor growth of SNU-739 cells with control  and ZNF706 knockdown in nude mice. Pictures of the removed tumors. **I** The final tumors were weighed and plotted. **J** The tumor volume was measured and plotted (n = 6). All data were present as mean ± SD and *P* values were determined by Student *t*-test and one-way ANOVA (**B**, **C**, **J**). **P < 0.01, ***P < 0.001.

To determine the cellular function of ZNF706, we first used two independent short hairpin RNAs (shRNAs) to knock down endogenous ZNF706 expression in HCC cells. Western blotting and qRT‒PCR were then performed to confirm the expression of ZNF706 in four HCC cell lines, and the results indicated that ZNF706 expression was obviously decreased (Fig. 2A and Supplemental Fig. 2A, B). The depletion of ZNF706 markedly decreased the growth of SNU-739, BEL-7404, SNU-368 and LM3 cells, as determined by Cell Counting Kit-8 (CCK-8) assays (Fig. 2B and Supplemental Fig. 2D). Moreover, a colony formation assay was performed, and we observed that ZNF706 knockdown noticeably suppressed the anchorage-dependent growth of these four liver cancer cell lines (Fig. 2D and Supplemental Fig. 2E). Accordingly, the inhibitory effect from ZNF706 knockdown was related to the significant inhibition of proliferation in the four liver cancer cell lines, as determined by soft agar assays (Fig. 2F and Supplemental Fig. 2F). To further investigate the functional impacts of ZNF706, we generated cell lines stably transduced with an empty lentiviral vector or a lentiviral vector overexpressing ZNF706 (Fig. 2A and Supplemental Fig. 2C). Intriguingly, overexpression of ZNF706 promoted the growth of SNU-739 and LM3 cells, as indicated by CCK-8 assays (Fig. 2C). In addition, both the colony formation assays and soft agar assays further confirmed that ZNF706 overexpression facilitated the growth of SNU-739 and LM3 cells (Fig. 2E, G).

To further confirm whether ZNF706 knockdown inhibits HCC cell growth, SNU-739 cells with ZNF706 knockdown and the corresponding control cells were subcutaneously injected into nude mice. Consistent with our in vitro observations, the weight and volume of tumors formed from ZNF706 knockdown cells were clearly lower than those of xenograft-derived from control cells (Fig. 2H–J). Taken together, these results support the idea that ZNF706 might play an oncogenic role in the HCC progression.

### ZNF706 promotes HCC progression by regulating SLC7A11 expression

**Fig. 3:**
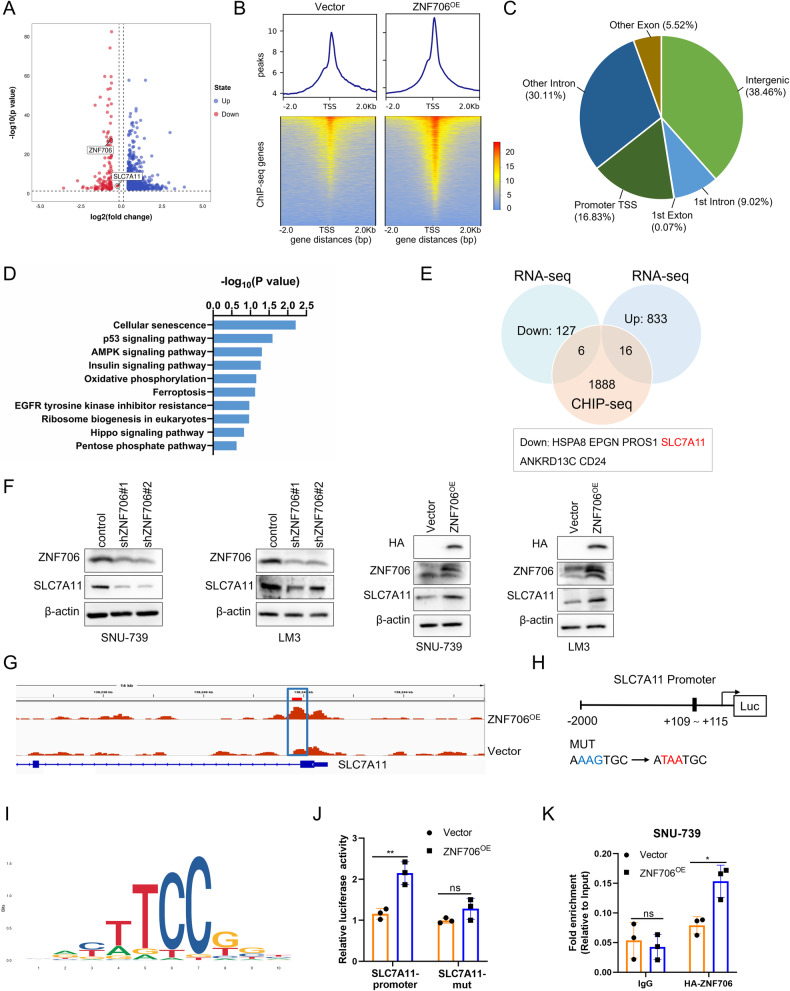
ZNF706 facilitates HCC progression through regulating SLC7A11 expression. **A** A volcano plot of RNA-seq in LM3-shZNF706 vs LM3-control cells showed the differentially expressed genes (|Log2 FC ≥ 0.3|, *p* < 0.05). Blue indicated upregulated, red indicated downregulated. **B** ZNF706 binding sites were rank-ordered according to ZNF706 ChIP-seq intensities in LM3-ZNF706 vs LM3-vector cells. **C** The genomic distribution of the transcriptional targets of ZNF706 was analyzed by ChIP-seq. **D** ChIP-seq analysis of GSEA showed the enriched pathway associated with ZNF706. **E** The overlapping genes were found by RNA-seq and ChIP-seq in LM3 cells. **F** The protein levels of SLC7A11 were measured in stably depleted or overexpressed ZNF706 HCC cells. **G** ChIP-seq analysis of the binding peaks of ZNF706 on the SLC7A11 genomic locus. **H** Schematic diagram of the binding site of ZNF706 on the SLC7A11 promoter region. SLC7A11 mutations shown in red part. **I** ChIP-seq analysis of the binding motifs of ZNF706 and SLC7A11. **J** Dual luciferase reporter assay driven by WT or mutant promoter was transiently transfected into HEK293T cells with or without ZNF706 overexpression. The relative luciferase activities were calculated as the ratio of firefly luciferase activity and Renilla luciferase activity. **K** ChIP-seq results were verified by ChIP‒qPCR with antibodies against HA-ZNF706 in SNU-739 cells with ZNF706 overexpression. All data were shown as mean ± SD. *P* values were determined by Student *t*-test. Ns nonsignificant, *P < 0.05, **P < 0.01.

To more accurately identify the molecular mechanisms underlying ZNF706-modulated biological processes, we performed RNA-seq analysis to explore the gene expression profiles of LM3 cells with stable ZNF706 knockdown and the corresponding vector control cells. The differentially expressed genes (DEGs) were identified as shown in Fig. 3A. Gene Ontology (GO) analysis revealed that multiple biological processes such as cell differentiation, oxidation‒reduction process, and protein phosphorylation, were enriched in the DEGs (Supplemental Fig. 3A). Subsequent Kyoto Encyclopedia of Genes and Genomes (KEGG) analysis revealed enrichment of the DEGs in the following pathways: PI3K-AKT signaling pathway, biosynthesis of amino acids and PPAR signaling pathway (Supplemental Fig. 3B). Moreover, gene set enrichment analysis (GSEA) suggested that the DEGs may function in ferroptosis (Supplemental Fig. 3E).

Although ZNF706 has been identified as a DNA-binding protein, no report showed the specific binding motif of ZNF706 on the genomic DNA. Therefore, we first performed ChIP-seq to search for specific DNA-binding sites of ZNF706 in LM3 cells by using antibodies against HA-ZNF706. According to the ChIP-seq analysis, we subsequently identified 11,350 ZNF706-specific binding peaks in the HA-ZNF706-overexpression group and 6713 binding peaks in the control group (Fig. 3B). We analyzed the distribution of these binding peaks across the genome and identified 1512 binding peaks associated with promoter transcription start sites (TSSs) (Fig. 3C). Furthermore, GSEA indicated that multiple pathways, including AMPK signaling pathway, ferroptosis and pentose phosphate pathway, were markedly deregulated (Fig. 3D). Moreover, integrated analysis of the RNA-seq and ChIP-seq data, we identified 22 overlapping genes closely associated with the expression pattern of ZNF706, among which SLC7A11 attracted our attention because of its ability to affect the intracellular redox balance and act as a key suppressor of ferroptosis (Fig. 3E).

A heatmap was generated to visualize the potential target genes involved in ferroptosis and influenced by ZNF706 knockdown (Supplemental Fig. 3C). Real-time PCR analysis was performed, and the results revealed that the mRNA expression of SLC7A11 was significantly downregulated in response to ZNF706 knockdown, while significantly upregulated in response to ZNF706 overexpression in SNU-739 and LM3 cells (Supplemental Fig. 3D, F, G). As further confirmation of these results, Western blot analysis revealed that ZNF706 knockdown markedly decreased the expression of SLC7A11 in SNU-739 and LM3 cells, and ZNF706 overexpression markedly increased the expression of SLC7A11 in these cells (Fig. 3F). Furthermore, the analysis of the TCGA database revealed that patients with high SLC7A11 expression had a lower survival rate than those with low SLC7A11 expression (Supplemental Fig. 3H). Moreover, by using the TCGA database, we observed a positive correlation between the ZNF706 and SLC7A11 expression in HCC samples (Supplemental Fig. 3I).

To explore the potential mechanism by which ZNF706 regulates SLC7A11, we identified the binding peaks of ZNF706 in the SLC7A11 promoter region by analyzing the ChIP-seq data (Fig. 3G). To further validate the above results, we performed a dual luciferase reporter assay and ChIP‒qPCR analysis. The results revealed a strong binding of ZNF706 to the SLC7A11 promoter (Fig. 3I-K). In summary, we are the first to identify the binding motifs of ZNF706, supporting the existence of a physical interaction and functional regulatory relationship between ZNF706 and SLC7A11 in HCC cells.

### Depletion of ZNF706 disrupts intracellular redox homeostasis by decreasing SLC7A11 expression

**Fig. 4:**
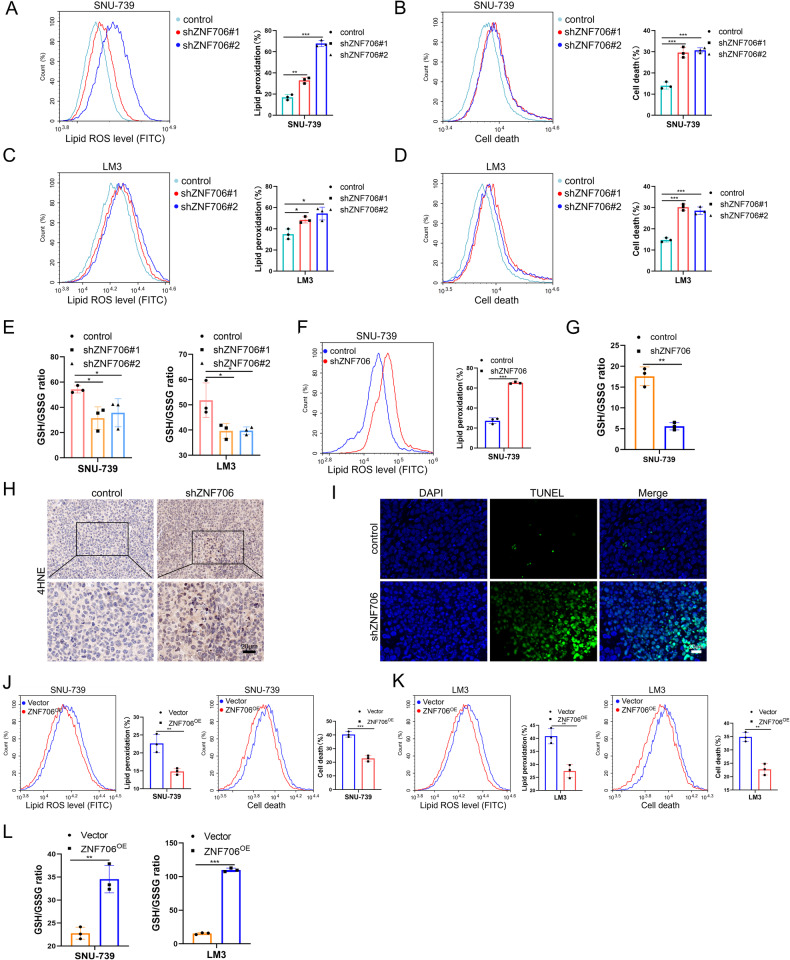
Knockdown of ZNF706 increased lipid ROS accumulation and disrupted redox homeostasis. **A**, **C** Lipid peroxidation was measured by flow cytometry in ZNF706-depleted SNU-739 and LM3 cells treated with erastin. **B**, **D** Cell death was examined by flow cytometry after treatment with erastin in ZNF706-depleted SNU-739 and LM3 cells. **E** The levels of glutathione were measured treated with erastin in SNU-739 and LM3 cells with stable ZNF706 knockdown. **F** The lipid peroxidation levels of subcutaneous tumor derived from ZNF706-depletion and control tumors were examined. **G** The expression of GSH derived from ZNF706-knockdown and control tumors were measured. **H** Immunohistochemical staining of 4HNE in tumor was examined. Scale bars: 20 μm. **I** Cell death was examined by immunofluorescent staining of TUNEL in tumors from ZNF706-depleted SNU-739 cells. Scale bars: 20 μm. **J**, **K** Lipid peroxidation and cell death were measured by flow cytometry after treatment with erastin in ZNF706-overexpressed SNU-739 and LM3 cells. **L** The relative expression of GSH was examined after treatment with erastin in SNU-739 and LM3 cells with ZNF706 overexpression. All data were shown as mean ± SD, and statistical significance was analyzed by Student *t*-test. *P < 0.05, **P < 0.01, ***P < 0.001.

Accumulating evidence has revealed that SLC7A11 plays critical roles in maintaining cell survival and redox homeostasis [23, 24]. We next sought to determine the potential role of ZNF706 in ferroptosis response. Strikingly, we observed that depletion of ZNF706 significantly increased erastin-induced lipid peroxidation in SNU-739 and LM3 cells (Fig. 4A, C). Moreover, a reduction in ZNF706 expression markedly facilitated erastin-induced cell death (Fig. 4B, D). It has been reported that SLC7A11 participates in regulating the uptake of extracellular cystine, an essential precursor for GSH biosynthesis, and we subsequently found that the level of GSH was decreased after ZNF706 knockdown in HCC cells (Fig. 4E). Furthermore, we found that subcutaneous tumors derived from ZNF706-depleted HCC cells had greater lipid peroxidation than tumors derived from control cells (Fig. 4F). Moreover, the level of GSH in ZNF706-knockdown tumors was decreased compared to that in control tumors (Fig. 4G). 4-Hydroxy-2-nonenal (4HNE) is an indicator of ferroptosis that can be used to evaluate lipid peroxidation [25]. Further examination of 4HNE by immunohistochemical (IHC) staining revealed that compared with control tumors, ZNF706-depleted tumors had increased 4HNE staining (Fig. 4H). Furthermore, the terminal deoxynucleotidyl transferase dUTP nick end labeling (TUNEL) assay revealed that ZNF706-knockdown tumors exhibited increased cell death compared with that in control tumors (Fig. 4I). In addition, ZNF706 staining was decreased in ZNF706-knockdown tumors (Supplemental Fig. 2G). Conversely, ZNF706 overexpression decreased lipid ROS levels and cell death and significantly increased the GSH level (Fig. 4J–L). In addition, knockdown of ZNF706 and SLC7A11 suppressed HCC cell proliferation (Supplemental Fig. 4A–D). Collectively, these data indicate that loss of ZNF706 suppresses HCC progression by repressing SLC7A11 expression and accelerating ferroptosis in HCC cells.

**Fig. 5:**
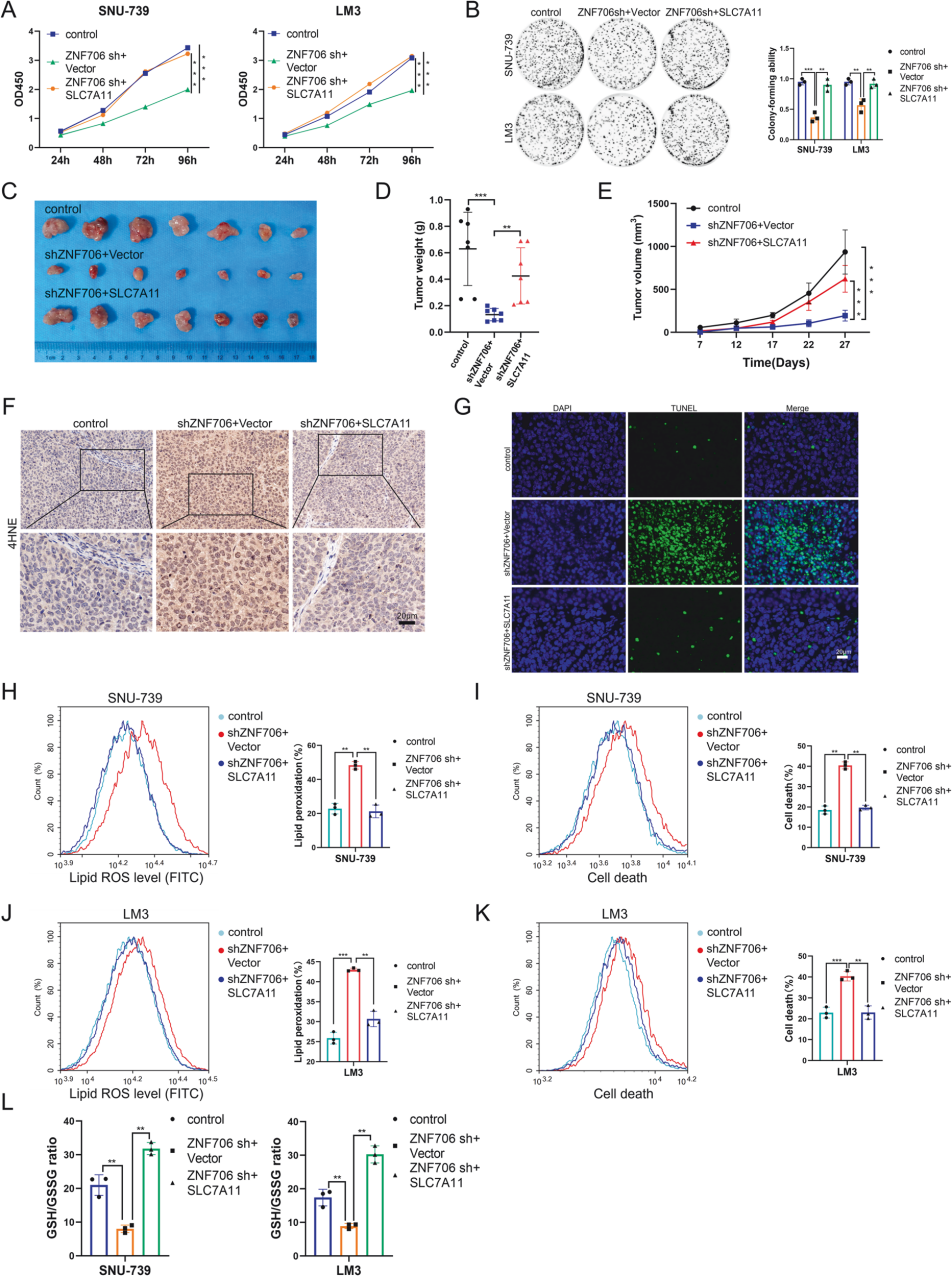
SLC7A11 overexpression partially rescues ZNF706 knockdown-induced disruption of redox homeostasis. **A**, **B** CCK-8, and colony formation assays were determined to measure the proliferative abilities of ZNF706-depleted SNU-739 and LM3 cells with SLC7A11 overexpression. **C** ZNF706-knockdown SNU-739 cells, with or without SLC7A11 overexpression, were subcutaneously injected into the nude mice. Pictures of the removed tumors. **D** The removed tumors were weighed and plotted. **E** The growth curves of subcutaneous tumors were monitored and plotted (n = 7). **F**, **G** Immunofluorescence staining of Immunohistochemical staining of 4HNE and TUNEL in tumor from ZNF706-knockdown SNU-739 cells, with or without SLC7A11 overexpression. Scale bars: 20 μm. **H**–**L** Lipid peroxidation, cell death, and GSH levels were measured after treatment with erastin in ZNF706-knockdown SNU-739 and LM3 cells with SLC7A11 reexpression. All results were present as mean ± SD. *P* values were determined by Student’s test and one-way ANOVA (**A**, **E**). **P < 0.01, ***P < 0.001.

To further confirm whether ZNF706 inhibits the expression of SLC7A11, we stably transfected ZNF706-depleted SNU-739 and LM3 HCC cells with an SLC7A11 overexpression construct or a control vector. The mRNA and protein levels of SLC7A11 were significantly increased after the restoration of SLC7A11 expression in ZNF706-knockdown HCC cells (Supplemental Fig. 4E, F). Interestingly, restoration of SLC7A11 expression in ZNF706-knockdown HCC cells partially reversed the inhibition of cell growth induced by ZNF706 knockdown, as determined by CCK-8 assays (Fig. 5A). Furthermore, colony formation assays and soft agar assays were performed, and the results confirmed that the growth ration of ZNF706-depleted SNU-739 and LM3 HCC cells was markedly increased by SLC7A11 overexpression (Fig. 5B and Supplemental Fig. 4G). In the in vivo assay, the ZNF706 knockdown-induced inhibition of xenograft growth was markedly reversed by restoration of SLC7A11 expression (Fig. 5C–E). Moreover, measurement of the 4HNE level and evaluation of cell death showed that compared with control tumors, ZNF706-knockdown tumors exhibited increased 4HNE staining and cell death; however, SLC7A11 overexpression significantly reduced these increases (Fig. 5F, G). In addition, IHC staining confirmed that ZNF706 and SLC7A11 expression was decreased in ZNF706-depleted tumors and that the level of SLC7A11 expression was increased after restoration of SLC7A11 expression (Supplemental Fig. 4H).

Further examination of lipid peroxidation revealed that the restoration of SLC7A11 expression in ZNF706-knockdown HCC cells inhibited erastin-induced lipid peroxidation (Fig. 5H, J). Notably, restoration of SLC7A11 in ZNF706-knockdown HCC cells significantly decreased erastin-induced cell death (Fig. 5I, K). Moreover, restoration of SLC7A11 expression in SNU-739 and LM3 HCC cells with stable knockdown of ZNF706 increased the level of GSH (Fig. 5L). Taken together, these findings reveal that SLC7A11 is essential for ZNF706-mediated malignant progression of HCC and responsiveness to ferroptosis.

### ZNF706 is transcriptionally activated by MYC

**Fig. 6:**
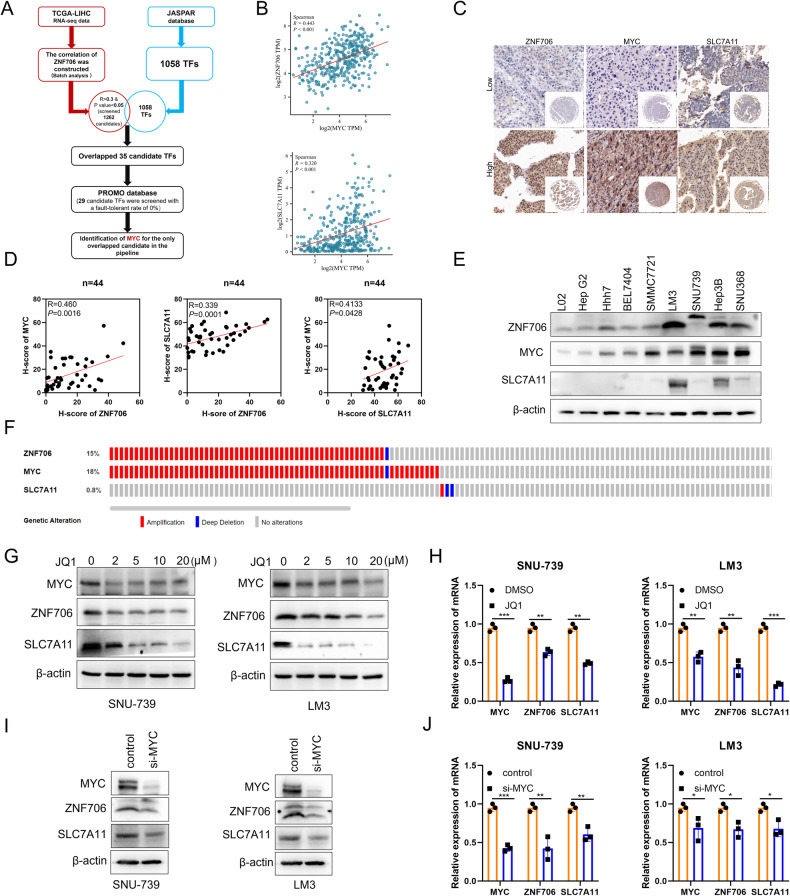
Coordinated expression of ZNF706, MYC, and SLC7A11 in liver cancer. **A** Transcription factor prediction for ZNF706 regulation using the TCGA, JASPAR, and PROMO databases. **B** The expression of MYC was positive correlation with the expression of SLC7A11 and ZNF706 from TCGA database. **C** IHC examined the protein levels of ZNF706, MYC, and SLC7A11 in liver cancer tissue chips (n = 44). **D** The correlation of ZNF706, MYC, and SLC7A11 was analyzed by linear regression analysis. **E** Western blot measured the protein levels of ZNF706, MYC, and SLC7A11 in HCC cells. **F** Analysis of genetic alterations of ZNF706, MYC, and SLC7A11 in liver cancer from cBioPortal database. **G** The protein levels of MYC and SLC7A11 were examined after adding different concentrations of JQ1 in SNU-739 and LM3 cells. **H** The relative expression of MYC and SLC7A11 were determined by qRT‒PCR in SNU-739 and LM3 cells treated with JQ1. **I**, **J** Western blot and qRT‒PCR analysis showed the levels of MYC, ZNF706, and SLC7A11 using siRNA targeting MYC knockdown in HCC cells. All data were represented as mean ± SD and statistical significance was determined by Student’s test and by linear regression analysis (**B**, **D**). *P < 0.05, **P < 0.01, ***P < 0.001.

To investigate the upstream regulatory mechanism of ZNF706 expression in HCC cells, we explored potential transcription factors (TFs) that may regulate ZNF706 expression in the mRNA level. We first analyzed the expression pattern of JASPAR-provided 1058 TFs with the correlation of ZNF706 expression in the TCGA-LIHC dataset. It showed that the expression level of 35 TFs has the positive correlation with ZNF706 in HCC samples. Furthermore, we used the PROMO to rank the putative TFs which may regulate ZNF706. And in the end of this pipeline mining the possible TF(s) regulating ZNF706, we identified that MYC might be a transcriptional factor regulating the expression of ZNF706 (Fig. 6A). And more importantly, a significant positive correlation was observed between MYC and ZNF706 or SLC7A11 expression in HCC samples by analyzing the TCGA database (Fig. 6B).

And then, to further determine the relationships among ZN706, MYC, and SLC7A11 in the protein level, we performed IHC analysis on cancer tissue chips to examine their expression pattern. The result showed that the alteration of their expression is consistent with each other from the same samples (Fig. 6C). And more importantly, IHC score analysis showed that they had a positive correlation with each other in the tested samples (Fig. 6D). Moreover, we also tested the expression pattern of ZN706, MYC, and SLC7A11 in HCC cell lines. The result of Western blot showed that they had a similar expression pattern in the tested cell lines (Fig. 6E). Results consistent with these findings were obtained in our analysis of data from cBioortal platform (Fig. 6F).

**Fig. 7:**
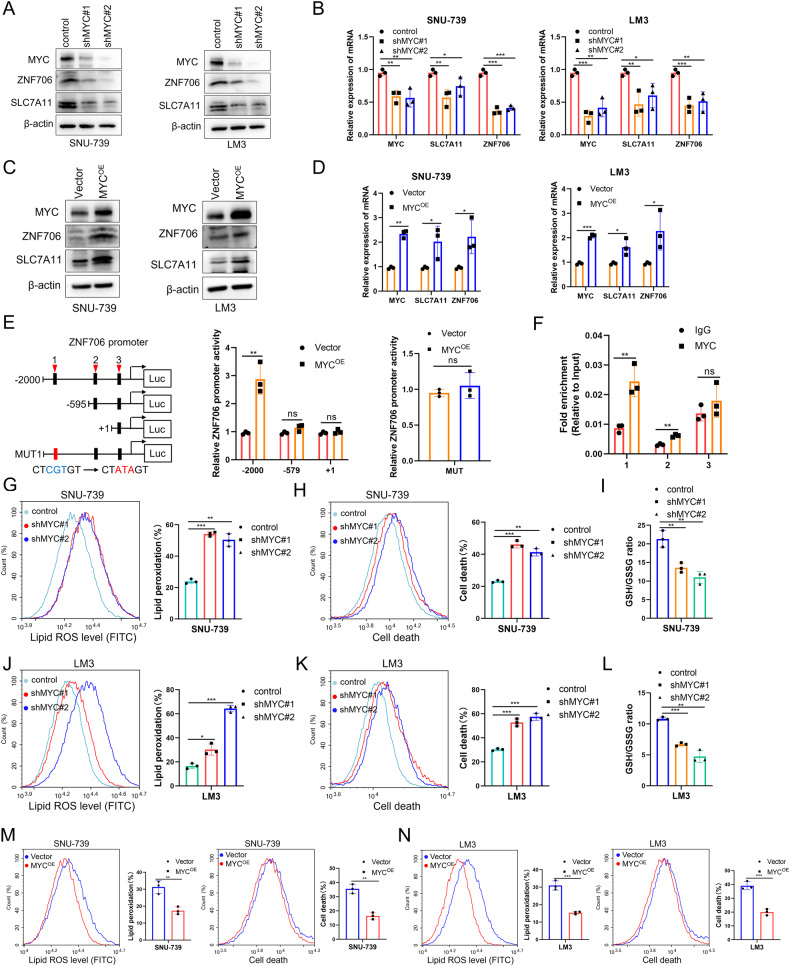
MYC transcriptionally activates ZNF706 and depletion of MYC promotes ferroptosis response by decreasing SLC7A11 expression. **A**, **B** The protein and mRNA levels of MYC, ZNF706, and SLC7A11 were measured after SNU-739 and LM3 HCC cells with stable depletion of MYC. **C**, **D** The levels of MYC, ZNF706, and SLC7A11 were examined in HCC cells with MYC overexpression. **E** Left: Schematic diagram of constructing serially truncated and mutant ZNF706 promoter. ZNF706 mutation displayed in red box. Right: The luciferase reporter activity of ZNF706 promoter was examined in HEK293T cells with or without MYC overexpression. **F** ChIP‒qPCR was determined with antibodies against MYC in SNU-739 cells with or without MYC overexpression. **G**, **J** Lipid peroxidation was examined after treatment with erastin in SNU-739 and LM3 cells with stable MYC knockdown. **H**, **K** Cell death was measured after treated with erastin in MYC-knockdown SNU-739 and LM3 cells. **I**, **L** The levels of GSH were determined after treatment with erastin in HCC cells with stably MYC-knockdown. **M**, **N** Lipid peroxidation and cell death were measured in MYC-overexpressed SNU-739 and LM3 cells treated with erastin. All data were represented as mean ± SD and statistical significance was determined by Student’s test. Ns nonsignificant, *P < 0.05, **P < 0.01, ***P < 0.001.

To investigate the hypothesized regulatory mechanism of MYC on the ZNF706 expression, we used a pharmacological inhibitor to block the activation of MYC in HCC cells. SNU-739 and LM3 cells were treated with different doses of MYC inhibitor JQ1. We found that administration of JQ1 obviously decreased the ZNF706 expression which was consistent with the inhibited expression of MYC in the protein level (Fig. 6G). And also, the mRNA expression of both MYC and ZNF706 was noticeably decreased (Fig. 6H). Moreover, we designed small interfering RNAs (siRNAs) targeting MYC to genetically inhibit MYC activation. Expectedly, the results revealed that the mRNA and protein expression of ZNF706 and SLC7A11 was obviously reduced (Fig. 6I, J). Similar results were obtained using two independent shRNAs to deplete MYC (Fig. 7A, B). To further verify the effect of MYC on the ZNF706 expression, we used lentiviral delivery system to overexpress MYC in SNU-739 and LM3 cells. The data revealed that overexpression of MYC markedly increased ZNF706 and SLC7A11 expression in HCC cells (Fig. 7C, D). To explore whether MYC binds to the ZNF706 promoter, we used the JASPAR database to predict and three putative MYC binding sites were shown on the promoter region of ZNF706. Furthermore, by using the dual luciferase reporter assay to test the activation of truncated ZNF706 promoter region, we found that the first binding site was the real responsive element recognized by MYC (Fig. 7E). To further demonstrate the occupancy of MYC in the ZNF706 promoter, we performed ChIP‒qPCR and confirmed that MYC physically interacts with the ZNF706 promoter region (Fig. 7F). Taken together, our results demonstrate that MYC transcriptionally activates ZNF706 in HCC cells.

### Overexpression of ZNF706 partially reverses MYC knockdown-induced disruption of the intracellular redox response

**Fig. 8:**
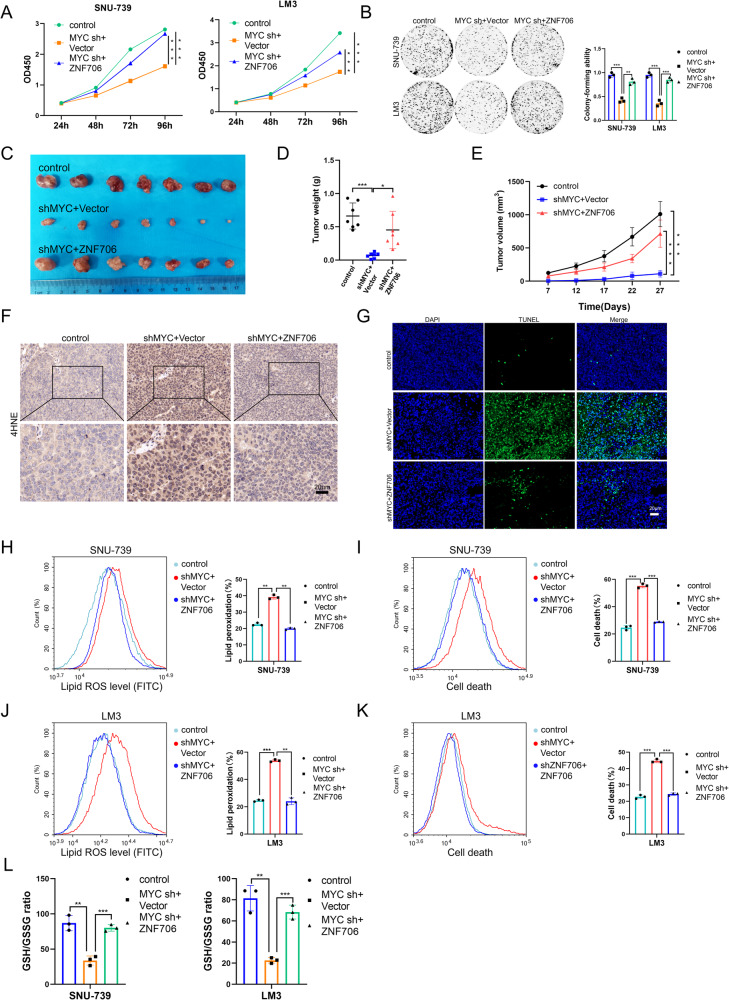
Restoration of ZNF706 partially rescues MYC knockdown-induced disruption of redox homeostasis. **A**, **B** Proliferative abilities of MYC-depleted SNU-739 and LM3 cells, with or without ZNF706 overexpression, were measured as judged by CCK-8 and colony formation assays. **C** MYC-knockdown SNU-739 cells with ZNF706 overexpression were subcutaneously injected into the nude mice. **D** The removed tumors were weighed. **E** The volumes of subcutaneous tumors were plotted (n = 7). **F**, **G** IHC staining and Immunofluorescence of 4HNE and TUNEL in tumor from MYC-knockdown SNU-739 cells with ZNF706 overexpression. Scale bars: 20 μm. **H**–**K** Lipid peroxidation and cell death were measured after treatment with erastin in MYC-depleted SNU-739 and LM3 cells with ZNF706 reexpression. **L** The levels of GSH were determined after treated with erastin in MYC-depleted SNU-739 and LM3 cells with or without ZNF706 reexpression. All results were shown as mean ± SD and statistical significance was determined by Student’s test and one-way ANOVA (**A**, **E**). *P < 0.05, **P < 0.01, ***P < 0.001.

Due to its involvement in reprogramming cellular metabolism, MYC is believed to be a hallmark of cancer progression that maintains the rapid proliferation of cancer cells [26]. A recent study reported that EGLN1 and MYC coordinately regulate lymphoid-specific helicase (LSH) expression to attenuate ferroptosis, indicating that MYC can be an important regulator of oxidative stress, which is involved in ferroptosis [27]. To clarify the potential role of MYC in ferroptosis in the context of HCC, we treated SNU-739 and LM3 cells with JQ1 to inhibit MYC expression and found that inhibition of MYC expression significantly increased erastin-induced ROS production and cell death (Supplemental Fig. 5A, B). Meanwhile, we also obtained similar results in HCC cells with shRNA-induced knockdown of MYC (Fig. 7G, H, J, K). Conversely, stable overexpression of MYC decreased lipid ROS production and cell death (Fig. 7M, N). We then measured the level of GSH and found that it was reduced after MYC depletion and increased after overexpression of MYC (Fig. 7I, L, and Supplemental Fig. 5C, D). Furthermore, we observed that double knockdown of ZNF706 and MYC inhibited HCC cell proliferation in vitro (Supplemental Fig. 6A–D). To deeply explore the underlying mechanism of MYC-regulated redox homeostasis, MYC-depleted SNU-739, and LM3 HCC cells were rescued by ZNF706 overexpression. The transfection efficiency was confirmed, and the level of ZNF706 was significantly increased after restoration of ZNF706 expression in MYC-depleted HCC cells (Supplemental Fig. 5E, F). Functionally, we observed that restoration of ZNF706 expression facilitated the MYC-induced inhibition of cell growth, as determined by CCK-8 and colony formation assays (Fig. 8A, B). In addition, subcutaneous tumors derived from cells with MYC-knockdown and ZNF706 overexpression grew faster than tumors derived from cells with only MYC knockdown (Fig. 8C–E). Compared with control tumors, MYC-knockdown tumors exhibited increased 4HNE staining and cell death; however, restoration of ZNF706 expression significantly inhibited these increases (Fig. 8F, G). Further analysis of lipid peroxidation and cell death revealed that ZNF706 restoration in MYC-knockdown HCC cells attenuated erastin-induced lipid peroxidation and cell death (Fig. 8H–K). Moreover, ZNF706 overexpression in MYC-knockdown HCC cells increased the GSH level (Fig. 8L). These observations indicate that ZNF706 is essential for MYC-mediated intracellular redox homeostasis.

### Knockdown of ZNF706 sensitizes HCC cells to Sorafenib

**Fig. 9:**
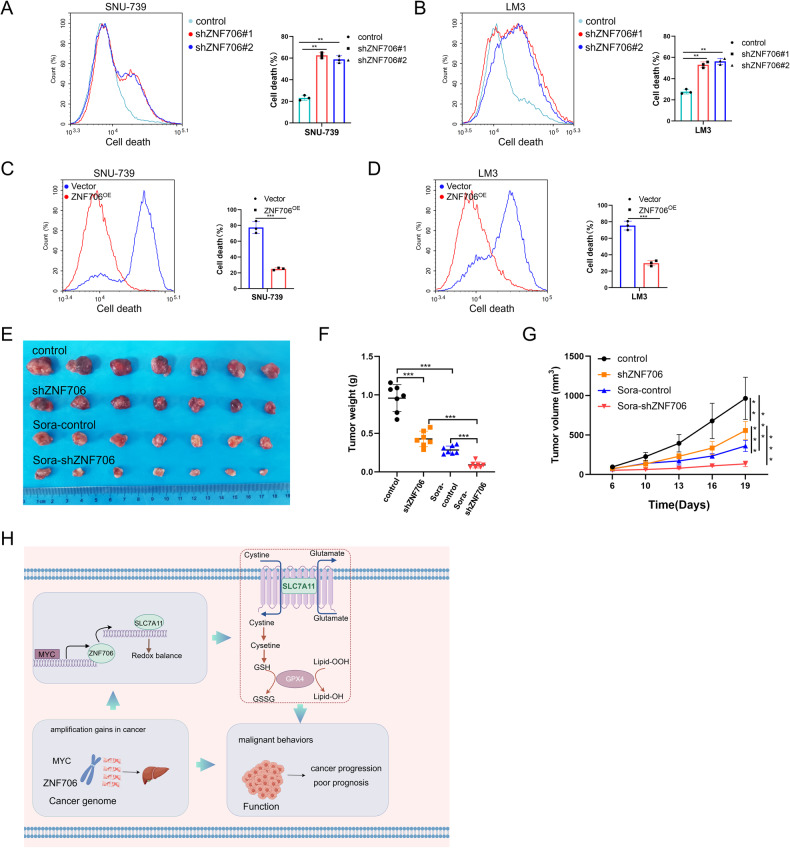
Depletion of ZNF706 sensitizes HCC cells to Sorafenib. **A**, **B** Cell death was examined by flow cytometry after treatment with Sorafenib in ZNF706-knockdown SNU-739 and LM3 cells. **C**, **D** Cell death was measured by flow cytometry after treatment with Sorafenib in ZNF706-overexpressed SNU-739 and LM3 cells. **E** ZNF706-knockdown and control SNU-739 cells were subcutaneously injected into the nude mice. The tumor volume reached 50–100 mm^3^, nude mice were administrated with 50 mg/kg Sorafenib every two days by gavage. **F** Tumor weight of nude mice was recorded. **G** The tumor volume was monitored and plotted (n = 7). **H** A schematic model for the MYC-ZNF706-SLC7A11 axis mediating HCC progression and redox balance. All data were represented as mean ± SD and statistical significance was determined by Student’s test and one-way ANOVA (**G**). **P < 0.01, ***P < 0.001.

Sorafenib, a multitarget tyrosine kinase inhibitor (TKI), is used as a first-line treatment for patients with late-stage HCC [28]. Accumulating evidence has revealed that attenuating oxidative stress protects cancer cells from the harmful effects of chemotherapy and radiotherapy [29]. Therefore, we sought to determine whether knockdown of ZNF706 sensitizes cancer cells to Sorafenib. It was showed that inhibition of ZNF706 notably increased HCC cell death and sensitivity to Sorafenib (Fig. 9A, B and Supplemental Fig. 7A). Conversely, ZNF706 overexpression suppressed Sorafenib-induced HCC cell death (Fig. 9C, D and Supplemental Fig. 7B). In addition, lipid peroxidation was increased in ZNF706-knockdown cells treated with Sorafenib; however, Sorafenib-treated ZNF706-overexpressing cells exhibited decreased lipid peroxidation (Supplemental Fig. 7C–F). Furthermore, in a mouse xenograft model, we observed that subcutaneous tumors derived from ZNF706-knockdown cells were smaller than tumors derived from control cells and that Sorafenib weakly repressed the growth of tumors derived from control cells but noticeably inhibited the growth of tumors derived from ZNF706-knockdown cells (Fig. 9E–G). Therefore, our findings support the idea that inhibition of ZNF706 sensitizes cancer cells to Sorafenib.

## Discussion

In the present study, we identified the biological roles and regulatory mechanisms of ZNF706, a novel zinc finger nuclear protein, in advanced progression of human hepatocellular carcinoma. In particular, we demonstrated its role in the regulation of intracellular redox homeostasis and tumor growth in HCC. MYC is a key oncogenic transcriptional factor driving hepatocellular carcinoma progression, and a copy number amplification of chromosome 8q24, where it is located, is known to be a molecular event contributing to the elevated expression levels and enhanced activity of MYC in HCC [30]. ZNF706, the gene of interest in our study, is located in another copy number amplification region 8q22, within the adjacent chromosomal region of the MYC gene. Previous studies on the functional annotation of ZNF706 have been reported to be relatively scarce, and its role and mechanism in tumor progression are even more lacking. Here, by using a series of bioinformatics analyses and logical inference, together with the paradigm for the study of transcription factor, we demonstrated that MYC is an important upstream transcription factor of ZNF706. And MYC is also the first identified transcription factor regulating ZNF706. Our findings revealed the upstream regulatory mechanism of ZNF706 and establish a novel mechanistic link between MYC and its regulation on the malignant behaviors in HCC cells.

It has been previously reported that 8q22, a genomic amplified region, contains numerous functional genes that may be involved in the regulation of malignant behaviors in breast cancer. Through in-depth and meticulous functional experiments, it was demonstrated that MTDH, located in this local amplicon, plays a crucial oncogenic role in breast cancer chemotherapy resistance and distant organ metastasis [31]. Interestingly, some other molecules located in 8q22, including LAPTM4B and YWHAZ, were also identified as key functional molecules for anthracycline resistance and breast cancer recurrence [32]. More importantly, 8q22 was also reported to have a copy number amplification in HCC [33]. It has been revealed that GRHL2, a transcription factor located in 8q22, shows frequently high levels expression in HCC tissues and promotes tumor growth in HCC [34]. However, the functional study in this local region with copy number variants in HCC cells has not been sufficiently investigated. What we identified in the present study is ZNF706, a novel zinc finger nuclear protein, being located within the chromosome 8q22 region with poorly functional annotation and mechanistic study. Here, we found that ZNF706 is frequently highly expressed in HCC tissues in comparison with the normal tissues. And the high level of ZNF706 expression is closely associated with a poor prognosis in the HCC patients, indicating that ZNF706 could be a potential biomarker in the clinic. By using gain- and loss- of functional experiments in vitro and in vivo, we observed the potential oncogenic effects of ZNF706 in HCC. And more importantly, we identified its role in the regulation of redox homeostasis in HCC cells. Mechanistically, by integrating data from ChIP-seq, RNA-seq, a promoter reporter assay, and ChIP-qPCR, we demonstrated that SLC7A11, an oxidative suppressor, is transcriptionally activated by ZNF706 in HCC cells. Furthermore, we confirmed a closely positive correlation among MYC, ZNF706, and SLC7A11 with each other in the mRNA and protein levels, indicating ZNF706 might be a critical hub to functionally link MYC to the maintenance of redox homeostasis in tumor cells by regulating SLC7A11 and consequently promotes advanced progression in HCC (Fig. 9H).

The maintenance of redox homeostasis is of paramount biological importance for cancer cell survival, proliferation, drug responsiveness, and even distant organ metastasis [35, 36]. Excessive accumulation of peroxides would lead to DNA damage, misfolding of functional proteins, or even cell death. Whereas, it has been demonstrated that the anti-oxidative molecules would scavenge the overloaded ROS species, prevent tumor cells from accumulated ROS-induced cell death, and even promote cancer metastasis. It has been commonly accepted that SLC7A11 plays an important role to promote tumor cell proliferation by inhibiting ROS accumulation in multiple types of cancer cells [37, 38]. As the gene encoding the cystine/glutamate antiporter, SLC7A11 mediates the uptake of cystine, a precursor for GSH biosynthesis. It was demonstrated to facilitate oncogenic KRAS-induced transformation in vitro and in vivo by mitigating oxidative stress [11]. Following the concept of ferroptosis, a lipid oxidation-induced new form of cell death, it was more widely accepted to by functionally associated with the regulation of SLC7A11. Previous studies have reported that transcriptional factors, such as NRF2, ATF4, SOX2 et al., affect ferroptosis responsibility by regulating the SLC7A11 expression [39, 40]. Here, we demonstrated that ZNF706 is a novel identified transcriptional factor regulating SLC7A11 and ferroptosis responsiveness in HCC cells.

In the clinical application, Sorafenib has been used the first-line drug for the treatment of HCC patients. And it has been demonstrated that oxidative stress-induced ferroptosis is one important mechanism attributing to Sorafenib-triggered antitumor effect [41]. Recently, SLC7A11-mediated Sorafenib resistance has been a matter with great concern. And our in vitro and in vivo study also showed that ZNF706 inhibition can significantly sensitize the response of HCC cells to the Sorafenib administration. And more importantly, our identification of a MYC-ZNF706-SLC7A11 regulatory axis provides a novel perspective to understand the development and progression of human hepatocellular carcinoma from the maintenance of redox homeostasis. Interestingly, it was recently reported that SLC7A11 drastically increases the expression of c-Myc through cysteine in cancer stem cells [42].

In summary, we present compelling evidence indicating that ZNF706-mediated regulation of SLC7A11 promotes the evasion of ferroptosis by increasing redox status. Our findings shed light on a critical oxidative stress evasion pathway and propose that blockade of the MYC-ZNF706-SLC7A11 axis could be a potential strategy in the treatment of HCC.

## Materials and methods

### HCC patient samples

All clinical HCC and matched adjacent normal tissues were acquired from the Department of Hepatic Surgery, Xijing Hospital (Xi’an, China), an affiliated hospital of Fourth Military Medical University. Fresh human HCC specimens in this study were promptly stored in liquid nitrogen after collection during surgery. The use of HCC samples was approved by the Ethics Committee of Xijing Hospital, and all patients provided written informed consent before participation. Our study was complied with the Declaration of Helsinki.

### Cell culture

The human HCC cell lines SNU-739, BEL-7404, SNU-368, and LM3 were obtained from the Cell Bank of the Chinese Academy of Sciences (Shanghai, China). The human embryonic kidney epithelial cell line HEK293T was obtained from the American Type Culture Collection (ATCC). SNU-739, BEL-7404, and SNU-368 cells were cultured in Roswell Park Memorial Institute (RPMI) 1640 medium (Gibco) supplemented with 10% fetal bovine serum (FBS; Gibco). LM3 cells and HEK293T cells were maintained in Dulbecco’s modified Eagle’s medium (DMEM; Gibco) supplemented with 10% FBS (Gibco). All cell lines were incubated under standard culture conditions at 37 °C with 5% CO_2_.

### Plasmid construction

To construct the plasmids expressing human sequences (pCDH-HA-ZNF706, and pCDH-HA-MYC), PCR-amplified human ZNF706 and MYC, cDNA sequences were inserted into the lentiviral vector pCDH-CMV-MCS-EF1-Puro digested with the restriction enzymes BamHI and EcoRI. The pLVX-HA-SLC7A11 plasmid were purchased from fenghbio (Hunan, China). pLKO.1-shZNF706 and pLKO.1-shMYC were generated by inserting shRNAs into the pLKO.1 vector. For construction of the ZNF706 luciferase promoter and the SLC7A11 luciferase promoter, different fragments of human ZNF706 promoter DNA and SLC7A11 promoter DNA were PCR amplified and inserted into the pGL3 enhancer vector digested with the restriction enzymes KpnI and NheI. All constructs were verified by DNA sequencing. The relevant primers are listed in Supplemental Table 1.

### Cell transfection and lentiviral transduction

To construct the SNU-739, BEL-7404, and SNU-368 and LM3 cell lines with stable ZNF706/MYC knockdown, pLKO.1-shZNF706 and pLKO.1-shMYC were cotransfected with packaging and envelope plasmids (psPAX2 and pMD2.G) into HEK293T cells. Viral supernatants were collected 48 h and 72 h after transfection and filtered through a 0.25 μM strainer, after which the infected cancer cells were selected with 2 µg/ml puromycin. To generate the ZNF706/MYC/SLC7A11-overexpressing cell lines, the lentiviral plasmids pCDH-HA-ZNF706, pCDH-HA-MYC, and pLVX-HA-SLC7A11 were cotransfected with the psPAX2 and pMD2.G plasmids into HEK293T cells.

### RNA extraction and qRT‒PCR

Total RNA was extracted from HCC tissues and cells using TRIzol Reagent (Invitrogen, USA) according to the manufacturer’s instructions. In brief, 1 µg of total RNA was reverse transcribed to cDNA with PrimeScript RT Master Mix (TaKaRa, Japan). Then, qRT‒PCR analysis was performed in triplicate on a Bio-Rad CFX96 instrument with a SYBR Premix Ex Taq Kit (TaKaRa, Japan). β-actin was used as the internal control, and the relative expression of each target gene was calculated by the 2^−ΔΔCt^ method. The primers used for PCR are listed in Supplemental Table 2.

### Cell viability assay

After trypsinization and centrifugation, the harvested cells were seeded into a 96-well plate (1000 cells/well). A Cell Counting Kit-8 (Yeasen, Shanghai) was used to measure cell viability. In brief, a mixture of medium and CCK-8 reagent was added to the wells, and the plate was incubated at 37 °C for 2 h. The absorbance of the wells was measured at 450 nm using a Bio-Rad multimode plate reader (Hercules, USA).

### Colony formation assays

For the colony formation assays, in brief, 500 cells were seeded into 6-well plates and continued to incubate for 10 days. The cells were fixed with 4% paraformaldehyde for 20 min and stained with crystal violet. The colonies were then counted.

### Soft agar assay

The soft agar assay was performed to examine cell survival in 3D culture. The 6-well plates were covered by the mixture of medium and 1% low-melting agarose. The collected cells were mixed with 0.6% low-melting agarose and seeded into 6-well plates. After 16 days, the spheres were photographed to acquire images.

### Transwell assay

For the transwell assay, 5 × 10^4^ cells were collected and seeded in serum-free medium into the upper chamber of a 24-well plate coated with or without Matrigel (Corning, USA), and medium supplemented with 10% FBS was added to the lower chamber. After 24 h, the medium was removed with a cotton swab, and the cells were fixed with 4% paraformaldehyde for 20 min and stained with crystal violet. Random fields of view were selected for imaging and cell counting.

### Immunofluorescence assay

Cells were seeded in confocal dishes, fixed with 4% paraformaldehyde for 30 min, and permeabilized with 0.2% Triton X-100. The cells were incubated with primary antibodies at 4 °C overnight, subsequently washed twice with cold PBS, and incubated with secondary antibodies for 1 h in the dark. The cells were counterstained with 4′,6-diamidino-2-phenylindole (DAPI; Thermo Fisher Scientific, USA). A Nikon ECLIPSE Ti2 confocal microscope was used to acquire immunofluorescence images.

### Cell death assay

Cells were seeded in a 6-well plate and treated with drugs for 48 h. The cells were then stained with propidium iodide (BD Bioscience, USA), and cell death was evaluated by microscopy and imaging. After the cells were trypsinized and centrifuged, cell death was further quantified by flow cytometry.

### GSH assay

Cells were seeded into a 6-well plate and treated with drugs for 48 h. Cell lysates were collected, and the cellular GSH concentration was measured with a Reduced Glutathione (GSH) Assay Kit (Beyotime, Shanghai, China) according to the manufacturer’s guidelines. The xenograft tissues were ground and were performed follow-up experiments.

### Luciferase reporter assay

For the luciferase reporter assay, in brief, cells were cotransfected with pGL3-ZNF706 promoter fragments, pRL-TK, and pCDH-MYC plasmid for 48 h. Similarly, pGL3-SLC7A11 promoter, pRL-TK, and pCDH-ZNF706 were also cotransfected with cells for 48 h. After the cell lysates were collected, the promoter activities were examined using a dual luciferase reporter assay detection kit (Promega, USA). Subsequently, firefly luciferase activity was normalized to the corresponding Renilla luciferase activity.

### Lipid peroxidation assay

Cells were seeded into a 6-well plate, treated with drugs for 3 h, and incubated with 5 μM C11-BODIPY (Thermo Fisher Scientific, USA) in the dark for 30 min at 37 °C. Then, the cells were harvested, resuspended in PBS, and analyzed using an ACEA NovoCyte flow cytometer. For xenograft tissues, we used collagenase to isolate individual cells and performed follow-up experiments.

### RNA sequencing

RNA-seq was performed by LC-BIO Biotech, Inc. (Hangzhou, China). In brief, total RNA from HCC cells was extracted with TRIzol Reagent, and the RNA was evaluated with a Bioanalyzer 2100 (Agilent, USA). Then, after determining the quality of the RNA samples, a sequencing library was constructed from RNA samples with an RNA integrity number (RIN) > 7.0. RNA-seq was performed on an Illumina NovaSeq™ 6000 system. The sample reads were mapped to the human reference genome using HISAT2 (https://daehwankimlab.github.io/hisat2/). Differentially expressed genes (DEGs) were identified by DESeq2 software with the following criteria: *P* < 0.05 and absolute fold change ≥0.3. GO and KEGG pathway enrichment analyses of the DEGs were performed via the LC Cloud platform (http://www.geneontology.org/, https://www.kegg.jp/kegg/).

### Chromatin immunoprecipitation sequencing

Approximately 2 × 10^7^ ZNF706-overexpressing HCC cells were collected and subjected to ChIP-seq analysis by LC-BIO Biotech. The cells were fixed with 1% formaldehyde for 10 min, 0.125 M glycine was added, and the mixture was incubated for 5 min to terminate the fixation reaction. Then, the cells were lysed to extract the DNA‒protein complexes from the nucleus, and the DNA was fragmented using sonication. Chromatin DNA precipitated with an antibody against HA-ZNF706 was subjected to purification. In-depth whole-genome DNA sequencing was then performed using a NovaSeq 6000 system. The raw sequencing data were filtered by Trimmomatic, mapped to the reference genome via STAR software, and further subjected to peak calling and motif analysis with MACS2 software and HOMER.

### Western blot analysis

Cells were washed with cold phosphate-buffered saline (PBS) and collected with radioimmunoprecipitation assay (RIPA) lysis buffer. The cell lysates were then centrifuged at 4 °C and 12,000 rpm for 15 min, after which a bicinchoninic acid (BCA) protein assay kit (Mishubio, Xian, China) was used to determine the protein concentration. Equal amounts of protein from the cell lysates were separated on 10% sodium dodecyl sulfate (SDS)-polyacrylamide gels and transferred onto nitrocellulose membranes. The membranes were incubated with primary antibodies at 4 °C overnight. Following incubation with secondary antibodies at room temperature for 1 h, protein signals were visualized with an enhanced chemiluminescence (ECL) kit (Mishubio, Xian, China).

### Antibodies and reagents

The following antibodies were used in this study: anti-HA (1:1000, Cell Signaling Technology, #3724), anti-SLC7A11 (1:1000, Affinity, #12509), anti-β-actin (1:2000, Sigma‒Aldrich, #1978), anti-MYC (1:2000, Abcam, #32072), and anti-4HNE (1:300, Biorbyt, #100588). A polyclonal antibody against ZNF706 was produced by Bioworld Technology (Nanjing, China) according to standard procedures, as follows. In brief, experimental animals were immunized with peptide-coupled carrier proteins as complete antigens, and antibodies were then isolated from positive animal serum using affinity chromatography. Erastin and JQ1 were obtained from Selleck (Houston, USA).

### Mouse xenograft assay

All 5-week-old male nude mice were purchased from Vital River Laboratory Animal Technology Co., Ltd. (Beijing, China). Then, 5 × 10^6^ SNU-739 cells were suspended in Matrigel (Corning, USA) and injected into subcutaneous tissue. After 7 days, an electronic caliper was used to measure the tumor size. The tumor volume was calculated according to the following equation: tumor volume (mm^3^) = (tumor length) × (tumor width)^2^/2. After 27 days, the mice were sacrificed, and the tumors were excised and weighed. The use of animal models in this study was approved by the Institutional Animal Care and Use Committee of Fourth Military Medical University.

### Statistical analysis

The data are presented in this study as the means ± standard deviations (SDs). According to the type of experiment, the significance of differences was analyzed by 2-tailed Student’s *t*-test or one-way analysis of variance (ANOVA) using GraphPad Prism 8. Correlations among ZNF706, MYC, and SLC7A11 expression were evaluated by linear regression analysis. A *P* value less than 0.05 was considered to indicate statistical significance. The statistical details are shown in the figure legends.

## Supplementary information

Supplementary materials

Original Data File

## Data Availability

The raw RNA-seq and the ChIP-seq data and processed expression matrix were uploaded to the GEO database under the accession numbers GSE246330 and GSE246331, respectively.
