# Supplementary material for: A novel MYC-ZNF706-SLC7A11 regulatory circuit contributes to cancer progression and redox balance in human hepatocellular carcinoma
Source: Cell Death Differ. 2024 Jun 11;31(10):1333–48. doi: 10.1038/s41418-024-01324-3 (PMC11445280; doi:10.1038/s41418-024-01324-3)
Supplement: Supplementary file 1 — Supplementary materials [file 41418_2024_1324_MOESM1_ESM.docx]

**Supplementary Figure 1. ZNF706 is up-regulated in human HCC and predicts poor prognosis of HCC patients**

A, B. Representative images of the distribution of ZNF706 were shown by immunofluorescent imaging in SNU-739 and LM3 cells. Scale bars: 50 μm. C. The location of ZNF706 was analyzed by nucleocytoplasmic separation experiment in SNU-739 cells. D. ZNF706 antibody was prepared according to the requirements of antibody design.


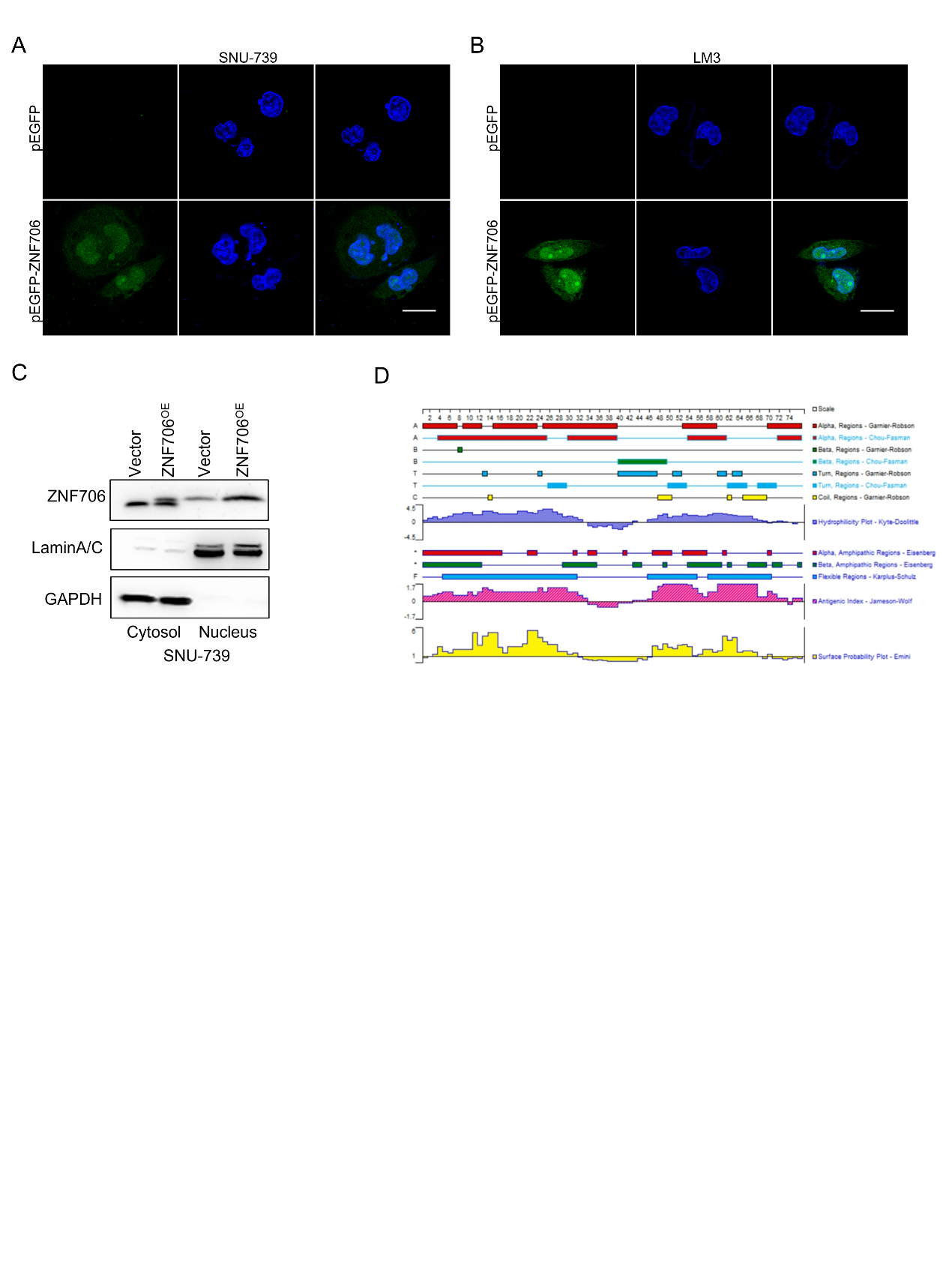


**Supplementary Figure 2. ZNF706 knockdown inhibits human HCC cells proliferation**

A. Western blot measured the levels of ZNF706 expression in SNU-739 and BEL-7404 cells with ZNF706 knockdown. B. Real-time PCR analysis of ZNF706 expression in SNU-739, BEL-7404, SNU-368 and LM3 cells. C. The mRNA expression of ZNF706 was examined in HCC cells with ZNF706 overexpression. D. Cell counting-8 kit (CCK-8) assay was used to examine the proliferative abilities of stably depleted ZNF706 cells. E, F. The proliferative abilities of stably depleted ZNF706 HCC cells were measured by plate clone assay and soft agar assay. Scale bars: 150 μm. G. Representative images of immunohistochemical staining of ZNF706 in xenograft tumors. Scale bars: 20 μm. **P* < 0.05, ***P* < 0.01, ****P* < 0.001.


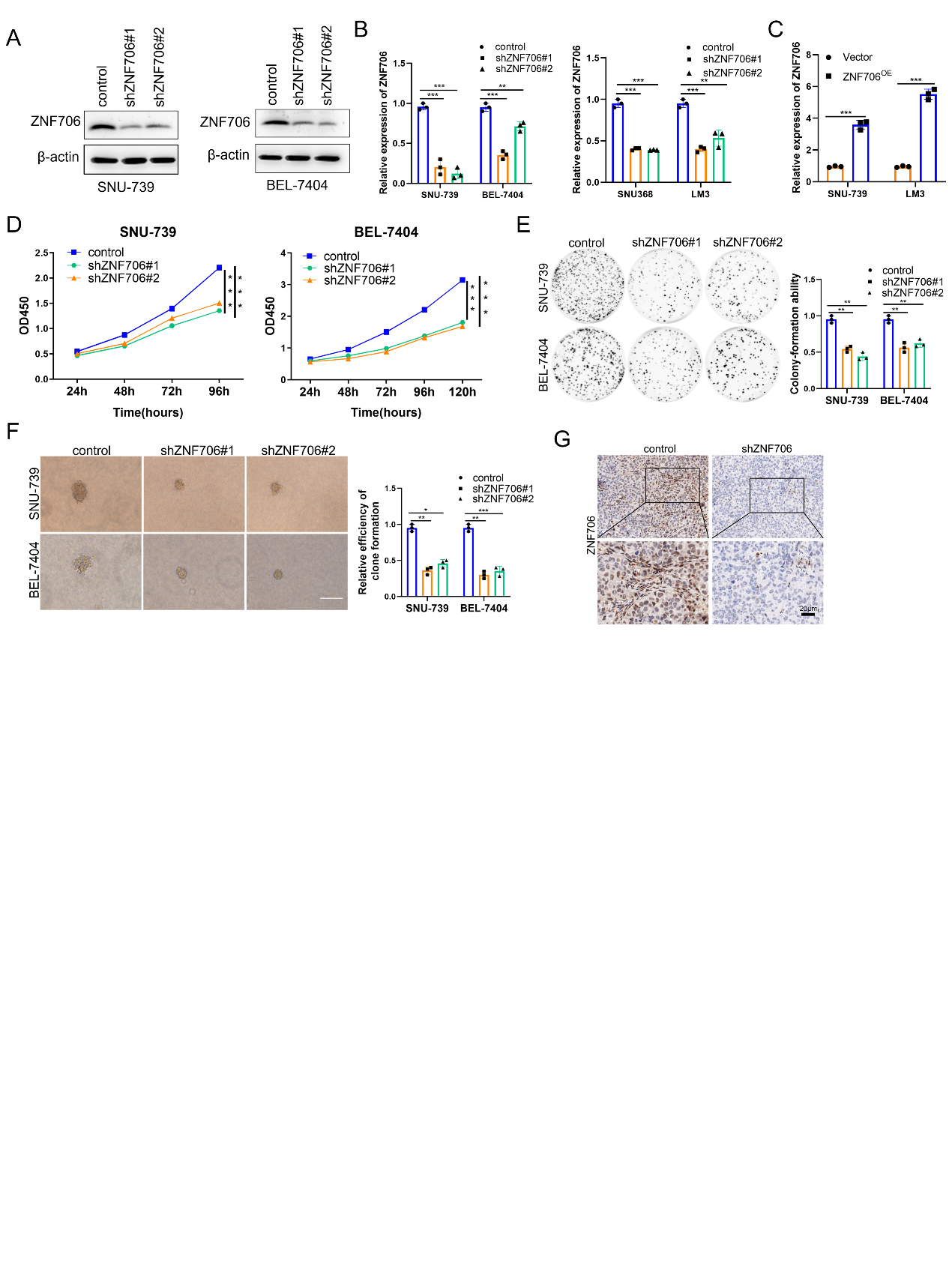


**Supplementary Figure 3. ZNF706 facilitates HCC progression through regulating SLC7A11 expression**

A. Go enrichment analysis displayed multiple biological processes by RNA-seq in LM3-shZNF706 vs LM3-control cells. B. Kyoto Encyclopedia of Genes and Genomes (KEGG) analysis showed enriched pathway related to ZNF706 by RNA-seq. C. Heatmap of RNA-seq depicted the levels of the most differentially expressed genes in ZNF706-associated ferroptosis pathway. D, F. qRT‒PCR assays were used to determine the levels of ferroptosis-related genes in SNU-739 and LM3 cells with ZNF706 knockdown. E. Gene set enrichment analysis showed the enrichment of gene sets related to ferroptosis pathway. G. Levels of SLC7A11 were examined in SNU-739 and LM3 cells with ZNF706 overexpression. H. High expression of SLC7A11 was associated with poor prognosis in liver cancer from TCGA database. I. The expression of ZNF706 was positive correlation with the expression of SLC7A11. Ns, nonsignificant, Ns, nonsignificant, **P* < 0.05, ***P* < 0.01, ****P* < 0.001.


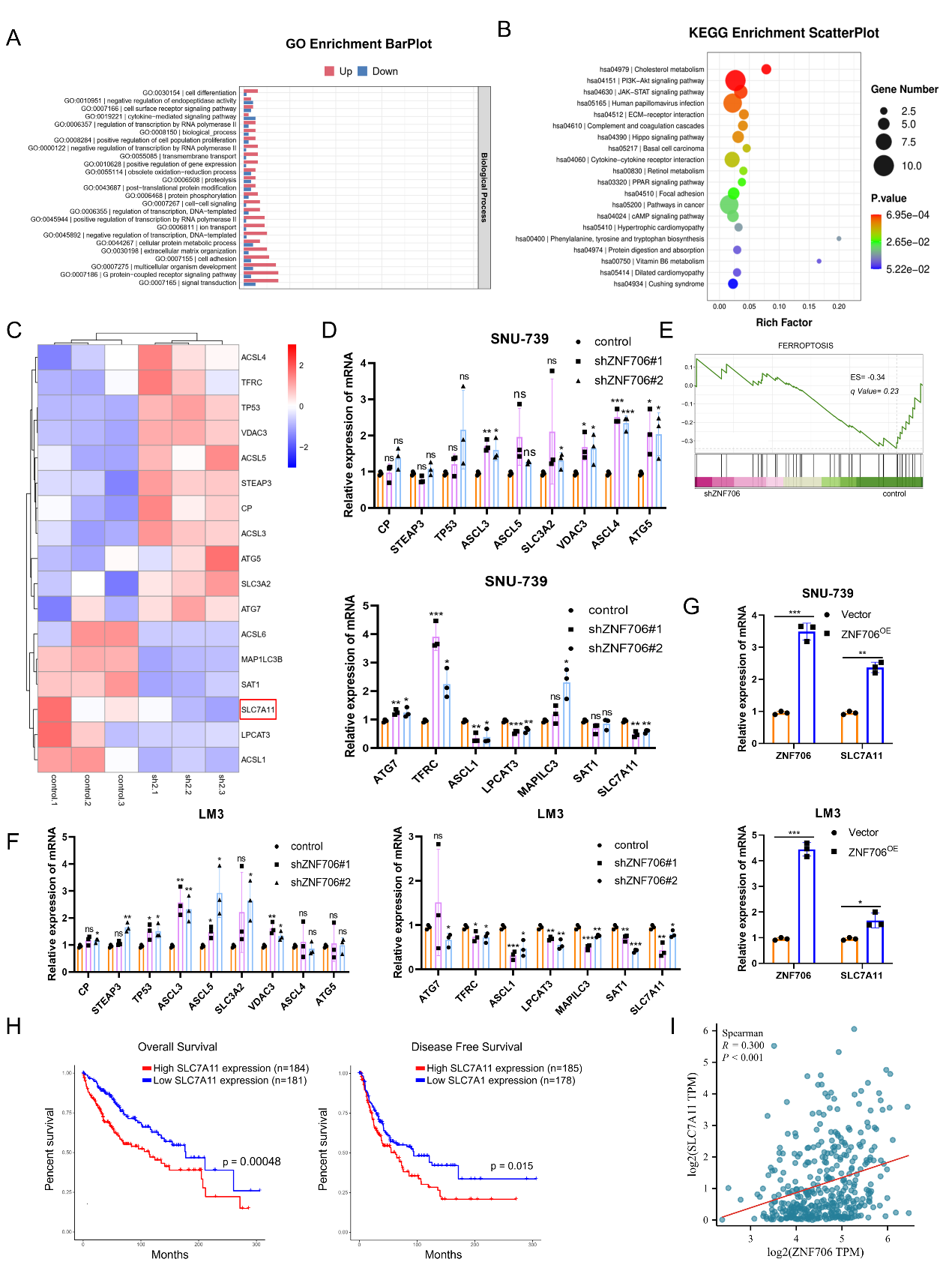


**Supplementary Figure 4. Knockdown of ZNF706 and SLC7A11 deterred HCC cell proliferation**

A, B. The protein and mRNA levels of ZNF706 and SLC7A11 were measured in ZNF706-knockdown HCC cells with siRNA targeting SLC7A11 knockdown. C, D. CCK-8, and colony formation assays were performed to examine the proliferative abilities of ZNF706 and SLC7A11 knockdown in SNU-739 and LM3 cells. E, F. The protein and mRNA levels of SLC7A11 and ZNF706 were measured in ZNF706-depleted SNU-739 and LM3 cells with SLC7A11 reexpression. G. Soft agar assay was applied to measure the proliferative ability in ZNF706-knockdown SNU-739 and LM3 cells with SLC7A11 overexpression. Scale bars: 150 μm. H. Representative images of immunohistochemical staining of ZNF706 and SLC7A11 proteins in xenograft tumors. Scale bars: 20 μm. Ns, nonsignificant, **P* < 0.05, ***P* < 0.01, ****P* < 0.001.


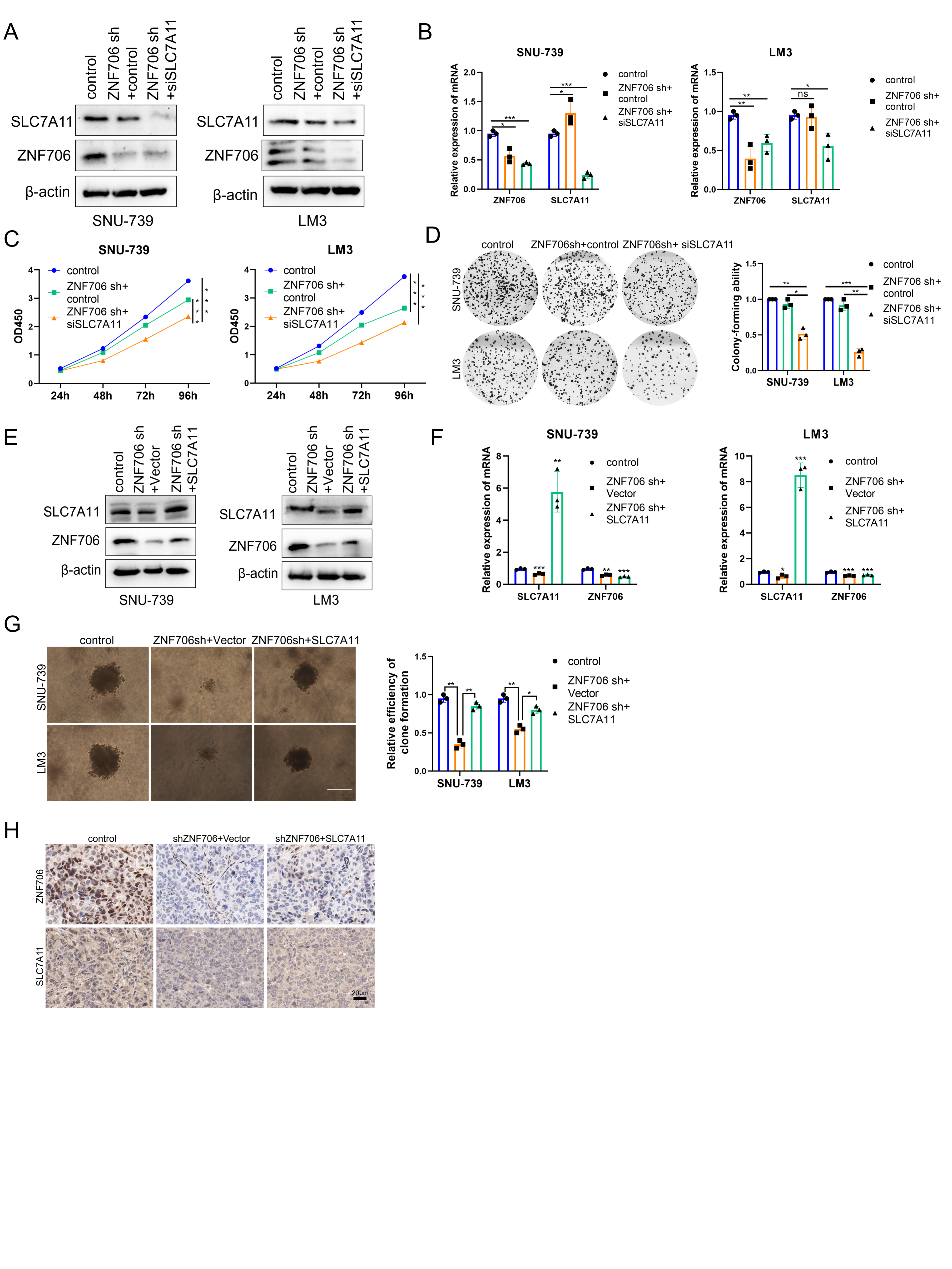


**Supplementary Figure 5. Knockdown of MYC facilitated ferroptosis response**

A, B. SNU-739 and LM3 cells treating with JQ1 to inhibit MYC expression examined lipid peroxidation and cell death by flow cytometry. C. The levels of GSH were measured after treatment with erastin in HCC cells using JQ1 to inhibit MYC expression. D. MYC-overexpressed SNU-739 and LM3 cells examined the expression of GSH after treatment with erastin. E. Western blot assays were performed to examine the levels of MYC, ZNF706 and SLC7A11 in MYC-knockdown SNU-739 and LM3 cells with ZNF706 reexpression. F. Real-time PCR assay was determined to measure the expression of MYC, ZNF706 and SLC7A11 in MYC-knockdown SNU-739 and LM3 cells with ZNF706 reexpression. **P* < 0.05, ***P* < 0.01, ****P* < 0.001.


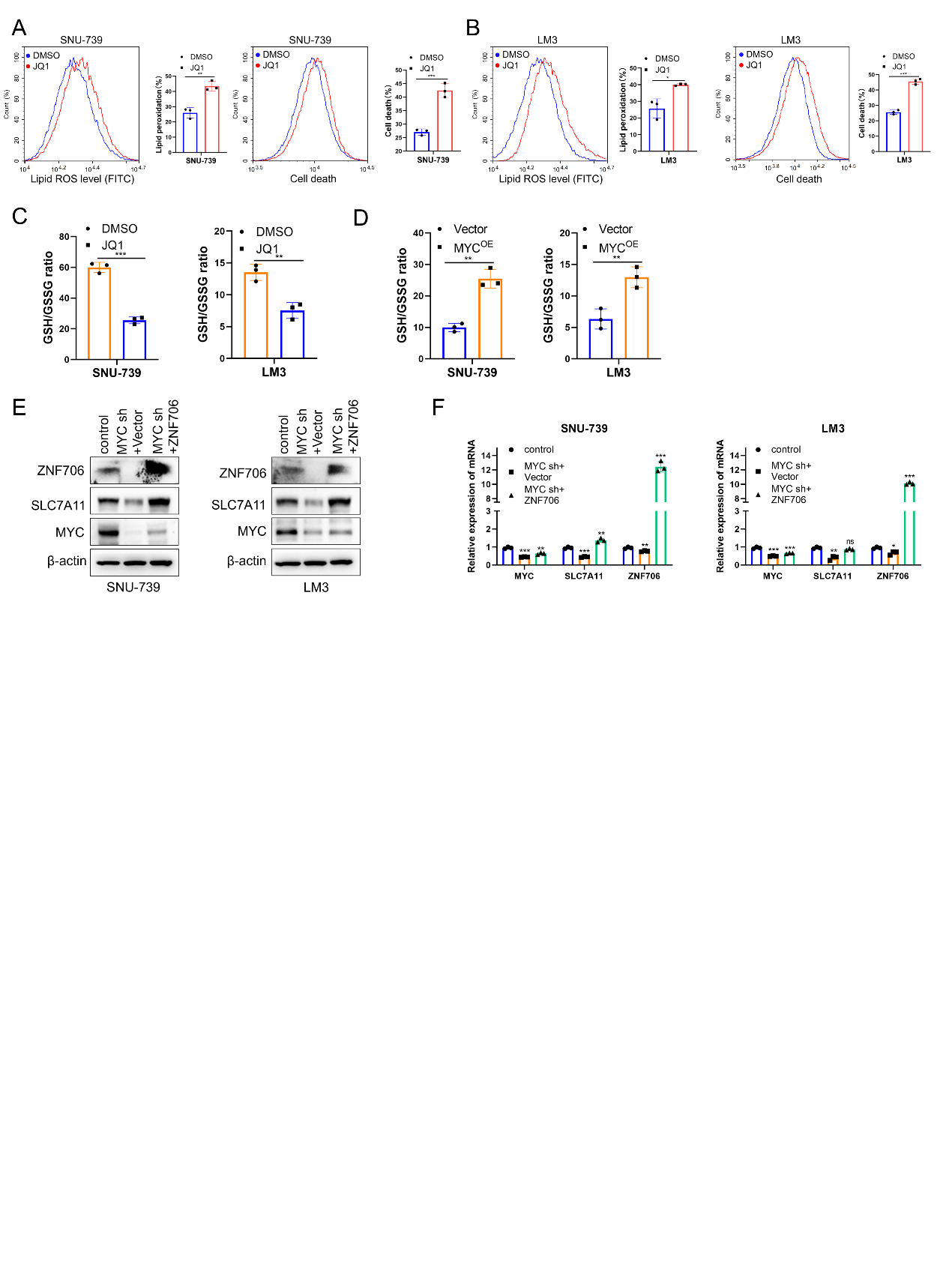


**Supplementary Figure 6. ZNF706 and MYC knockdown reduced HCC cell proliferation**

A, B. The protein and mRNA levels of ZNF706 and MYC were measured in ZNF706-knockdown HCC cells with siRNA targeting MYC knockdown. C, D. CCK-8, and colony formation assays were performed to measure the proliferative abilities of ZNF706 and MYC knockdown in HCC cells. **P* < 0.05, ***P* < 0.01, ****P* < 0.001.


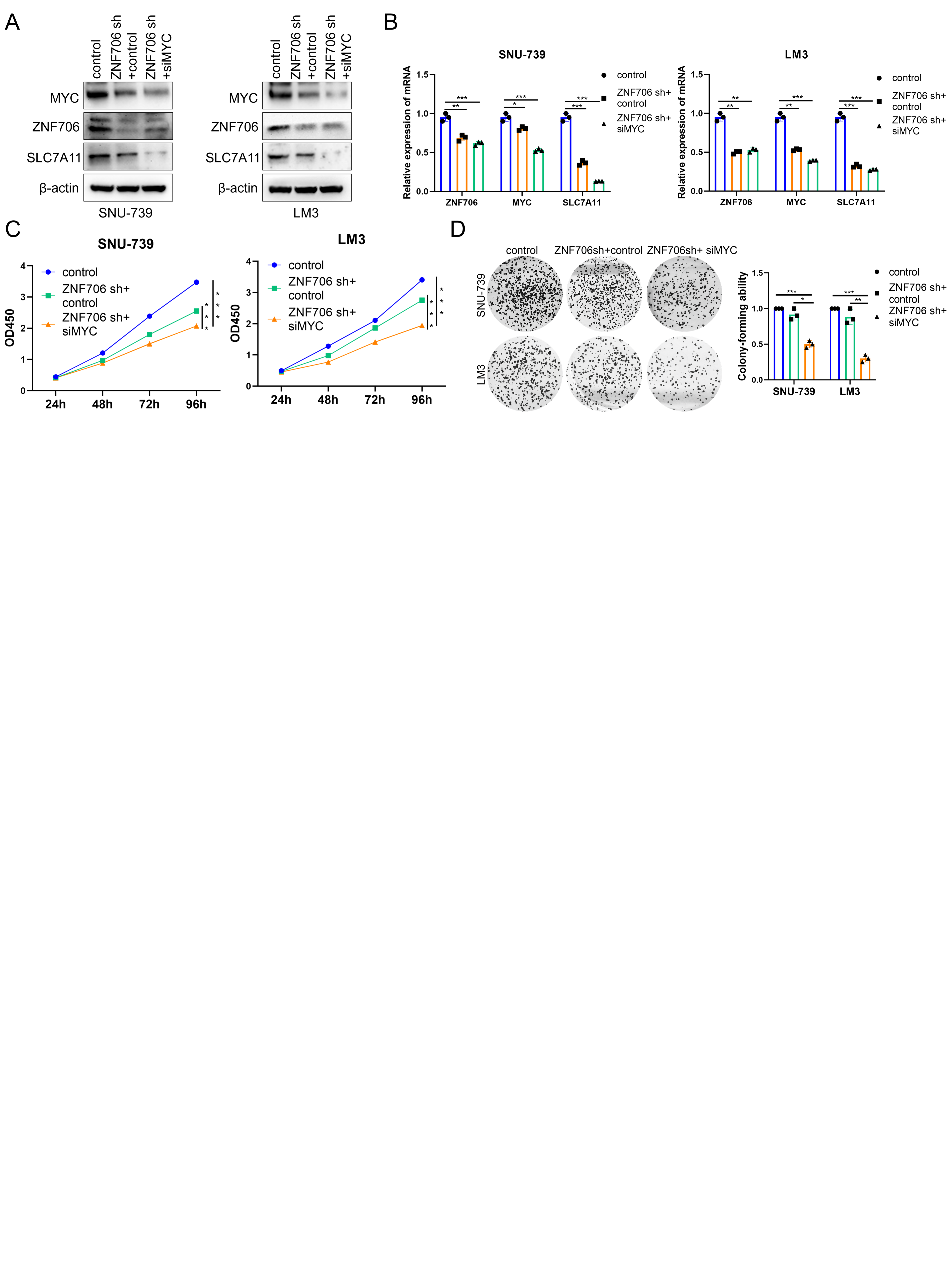


**Supplementary Figure 7 ZNF706 knockdown sensitizes HCC cells to Sorafenib**

A, B. Representative images of ZNF706-depleted SNU-739 and LM3 cells after treatment with Sorafenib were shown. C, D. Lipid peroxidation was measured by flow cytometry after treatment with Sorafenib in ZNF706-knockdown SNU-739 and LM3 cells. E, F. Lipid peroxidation was measured by flow cytometry after treatment with Sorafenib in ZNF706-overexpressed SNU-739 and LM3 cells. ***P* < 0.01, ****P* < 0.001.


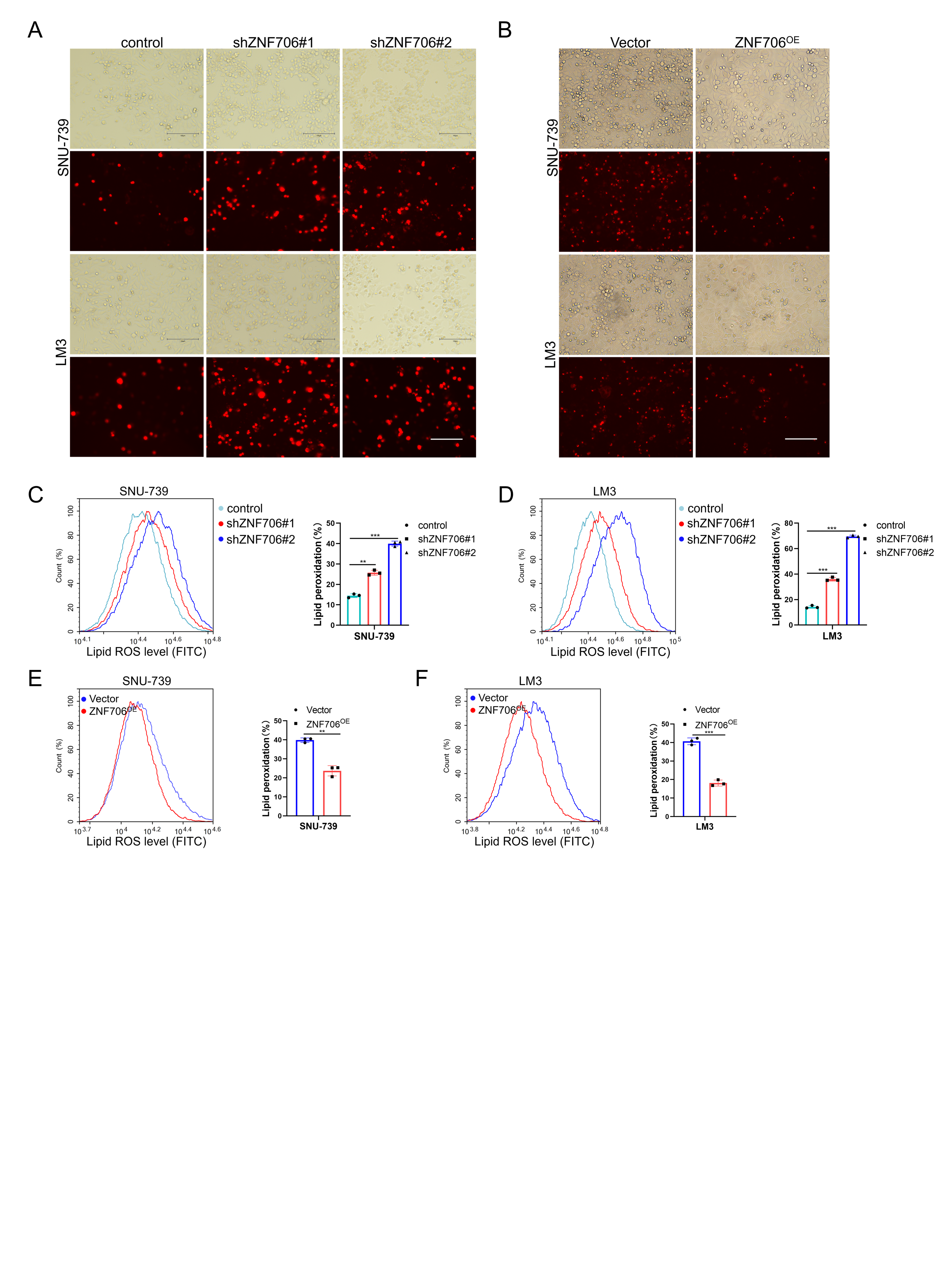


**Supplementary** **Materials and methods**

**Table S1. Primers used for generation of DNA constructs**

| **DNA construct** | **Primer sequences** | |
| --- | --- | --- |
| pCDH-ZNF706 | Forward primer | GAATTCATCGTAAGGGTGGACAA |
|  | Reverse primer | CTCGAGAGCTCTCAACCCCTGCA |
| pCDH-MYC | Forward primer | CTGGATTTTTTTCGGGTAGTGGAA |
|  | Reverse primer | TTACGCACAAGAGTTCCGTAG |
| pLVX-SLC7A11 | Forward primer | ATGGTCAGAAAGCCTGTT |
|  | Reverse primer | TCATAACTTATCTTCTTCTGGT |
| pGL3-enhancer-ZNF706 | Forward primer | CATTGTTTGGTGGGAATGGG |
|  | Reverse primer | ATCTAAAAACAGAAGGTGAAGC |
| pGL3-enhancer-ZNF706 MUT | Forward primer | TGTATTAGGCTCATATGCTA |
|  | Reverse primer | AGGGTAGCATATGAGCCTAA |
| pGL3-enhancer-SLC7A11 | Forward primer | TTCTTTCCCTGCCAACCTCT |
|  | Reverse primer | GTAGGAAGCAATTTGAGGGT |
| pGL3-enhancer-SLC7A11-MUT | Forward primer | GGCAGGAGATAATGCAGCTG |
|  | Reverse primer | CTTCAGCTGCATTATCTCCT |

**Table S2. Primers used for qRT‒PCR analysis**

| **Gene name** | **Forward primer** | **Reverse primer** |
| --- | --- | --- |
| ZNF706 | ACAAGGACATGACCAAAAGGC | GGAAGTGGAGTCTTAGGATGCTT |
| MYC | GGCTCCTGGCAAAAGGTCA | CTGCGTAGTTGTGCTGATGT |
| SLC7A11 | CCCACATTTCCTTCTTATCAACAG | GGCATCTCTCGCTTCATCTT |
| β-actin | CGGCACCACCATGTACCCTG | ACACGGAGTACTTGCGCTCA |
| CP | GGGCCATCTACCCTGATAACA | TTAAAGGTCCGATGAGTCCTGA |
| STEAP3 | CTCCCCGGAGGTCATCTTTG | TCTTGCTCTGTAGGGTTGCTC |
| TP53 | CAGCACATGACGGAGGTTGT | TCATCCAAATACTCCACACGC |
| ACSL3 | GCCGAGTGGATGATAGCTGC | ATGGCTGGACCTCCTAGAGTG |
| ACSL5 | CTCAACCCGTCTTACCTCTTCT | GCAGCAACTTGTTAGGTCATTG |
| SLC3A2 | TGAATGAGTTAGAGCCCGAGA | GTCTTCCGCCACCTTGATCTT |
| VDAC3 | TTGTACCGAACACAGGAAAGAAG | CCCAGCCATAGATGGTTGGTC |
| ACSL4 | CATCCCTGGAGCAGATACTCT | TCACTTAGGATTTCCCTGGTCC |
| ATG5 | AAAGATGTGCTTCGAGATGTGT | CACTTTGTCAGTTACCAACGTCA |
| ATG7 | CAGTTTGCCCCTTTTAGTAGTGC | CCAGCCGATACTCGTTCAGC |
| TFRC | ACCATTGTCATATACCCGGTTCA | CAATAGCCCAAGTAGCCAATCAT |
| ACSL1 | CCATGAGCTGTTCCGGTATTT | CCGAAGCCCATAAGCGTGTT |
| LPCAT3 | GGAGCTGAGCCTTAACAAGTT | CAAAGCAAAGGGGTAACCCAG |
| MAP1LC3B | GATGTCCGACTTATTCGAGAGC | TTGAGCTGTAAGCGCCTTCTA |
| SAT1 | ACCCGTGGATTGGCAAGTTAT | TGCAACCTGGCTTAGATTCTTC |
