# Supplementary figures and images for: A novel MYC-ZNF706-SLC7A11 regulatory circuit contributes to cancer progression and redox balance in human hepatocellular carcinoma
Source: Cell Death Differ. 2024 Jun 11;31(10):1333–48. doi: 10.1038/s41418-024-01324-3 (PMC11445280; doi:10.1038/s41418-024-01324-3)

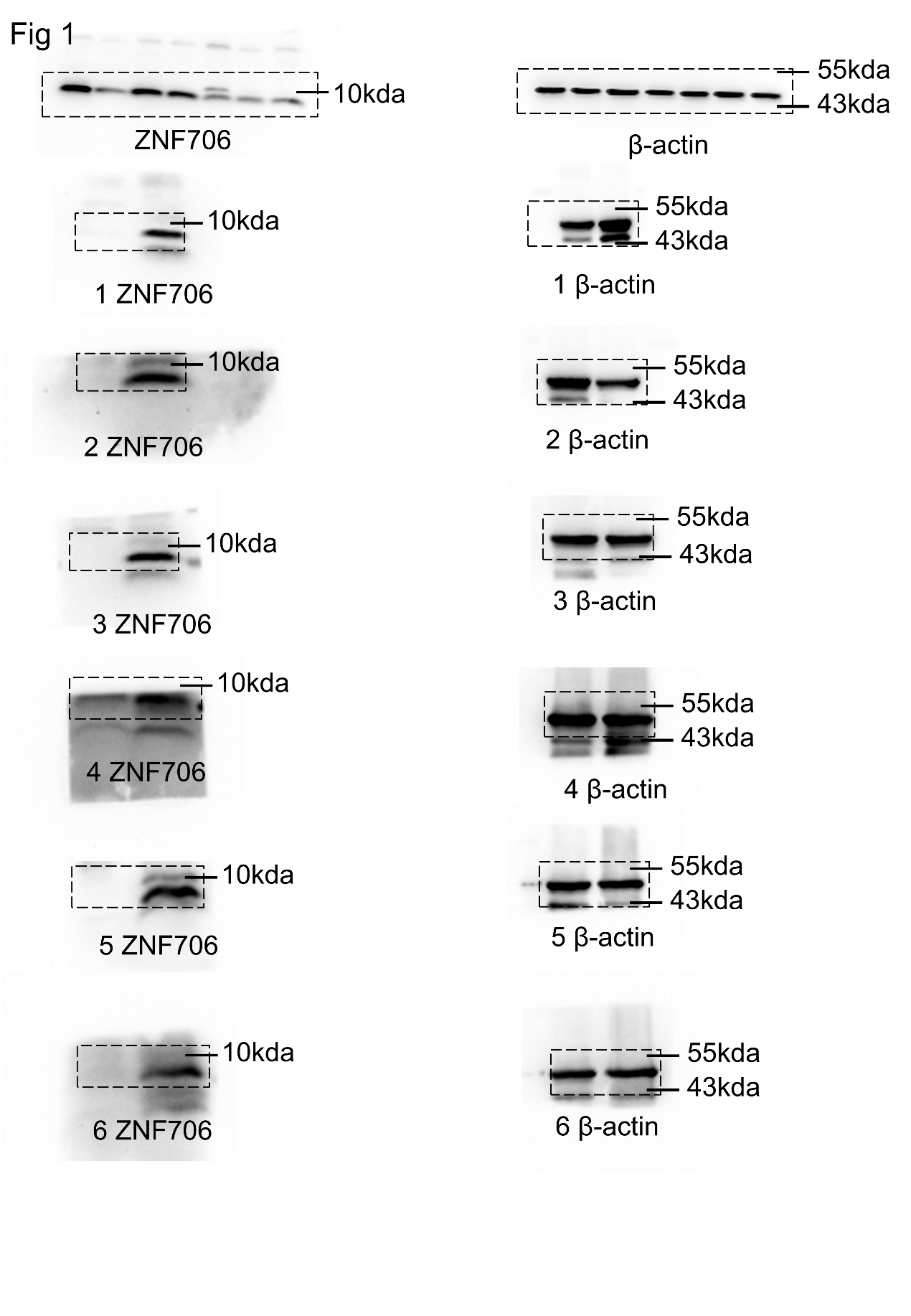

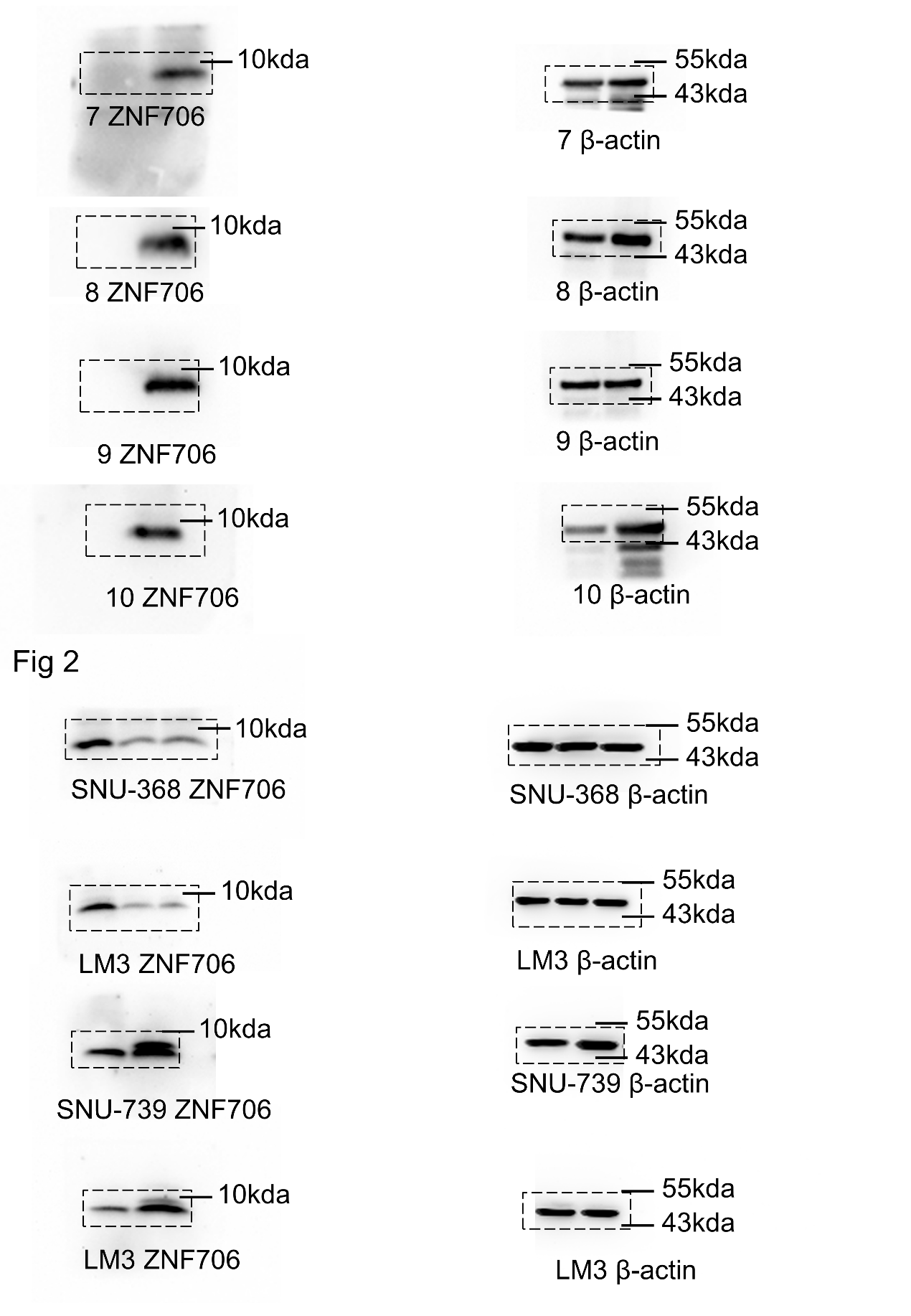

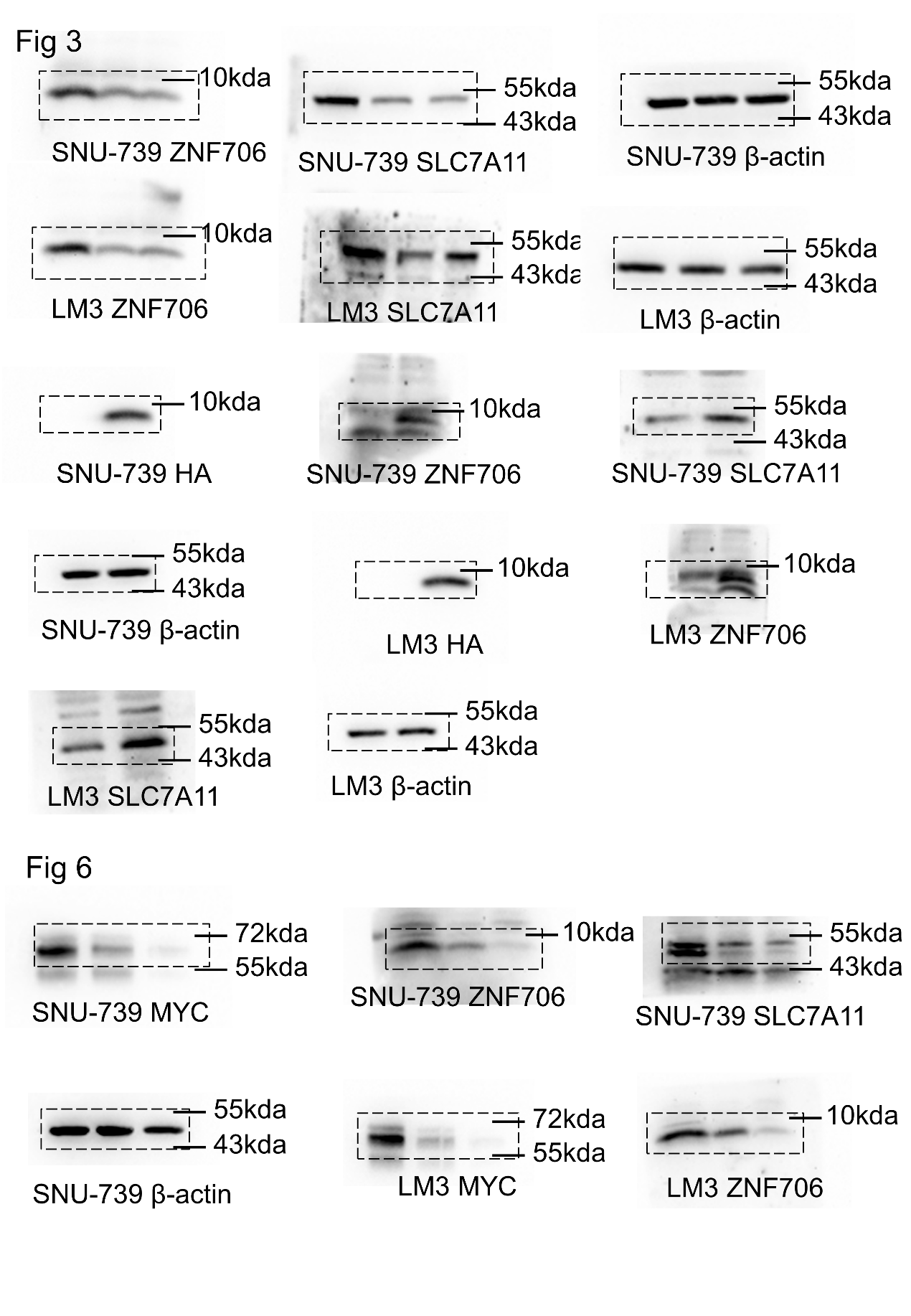

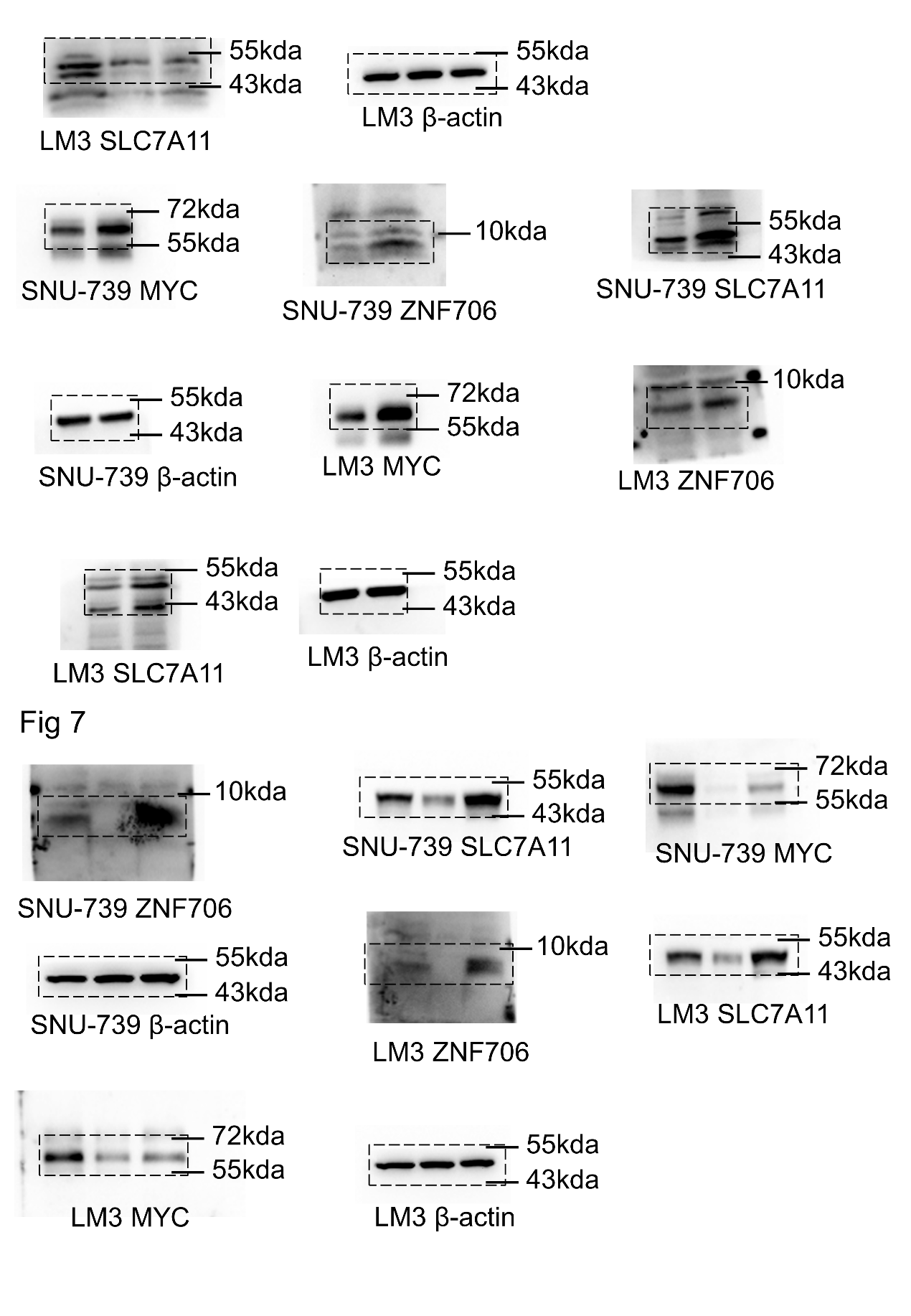

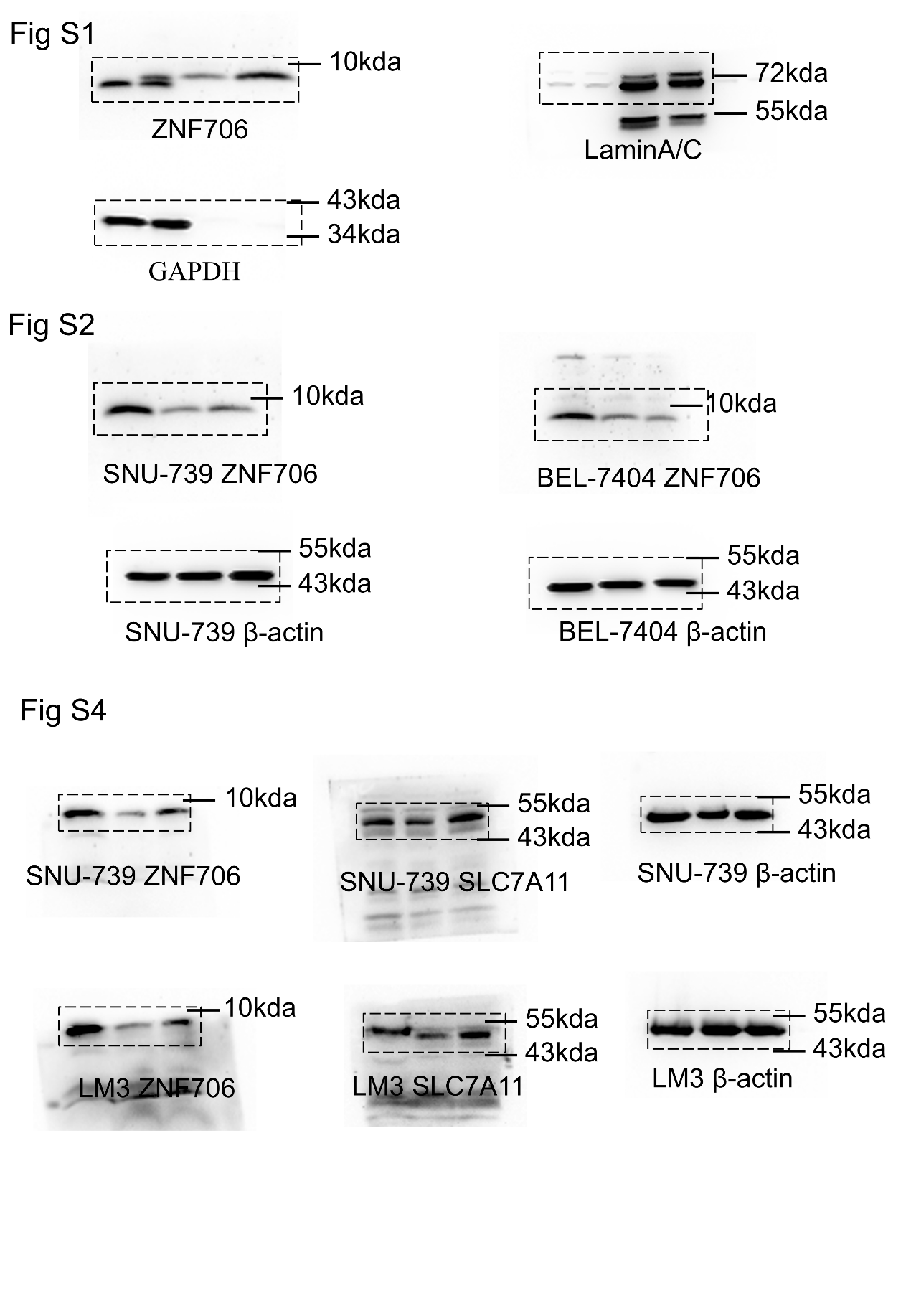

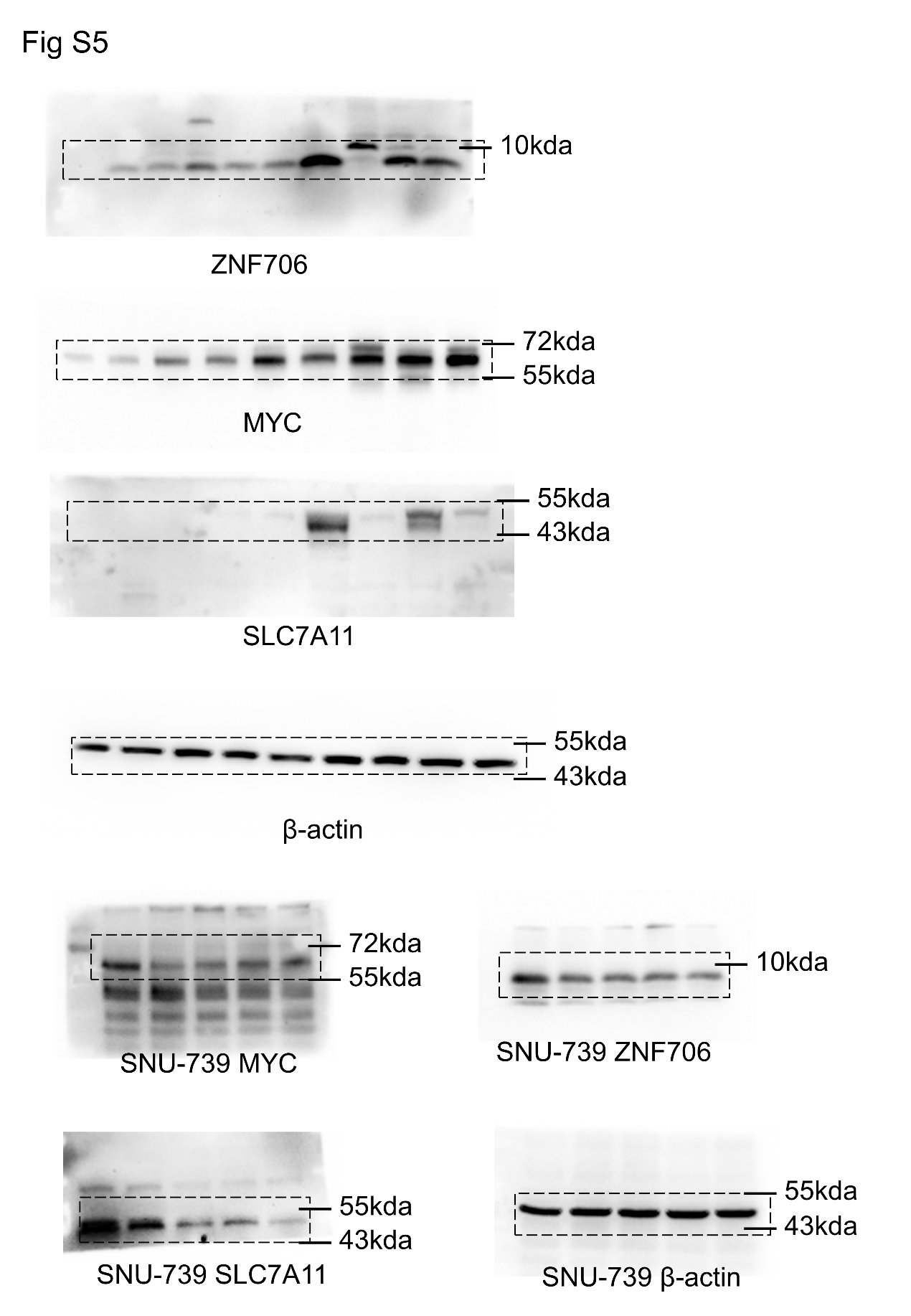

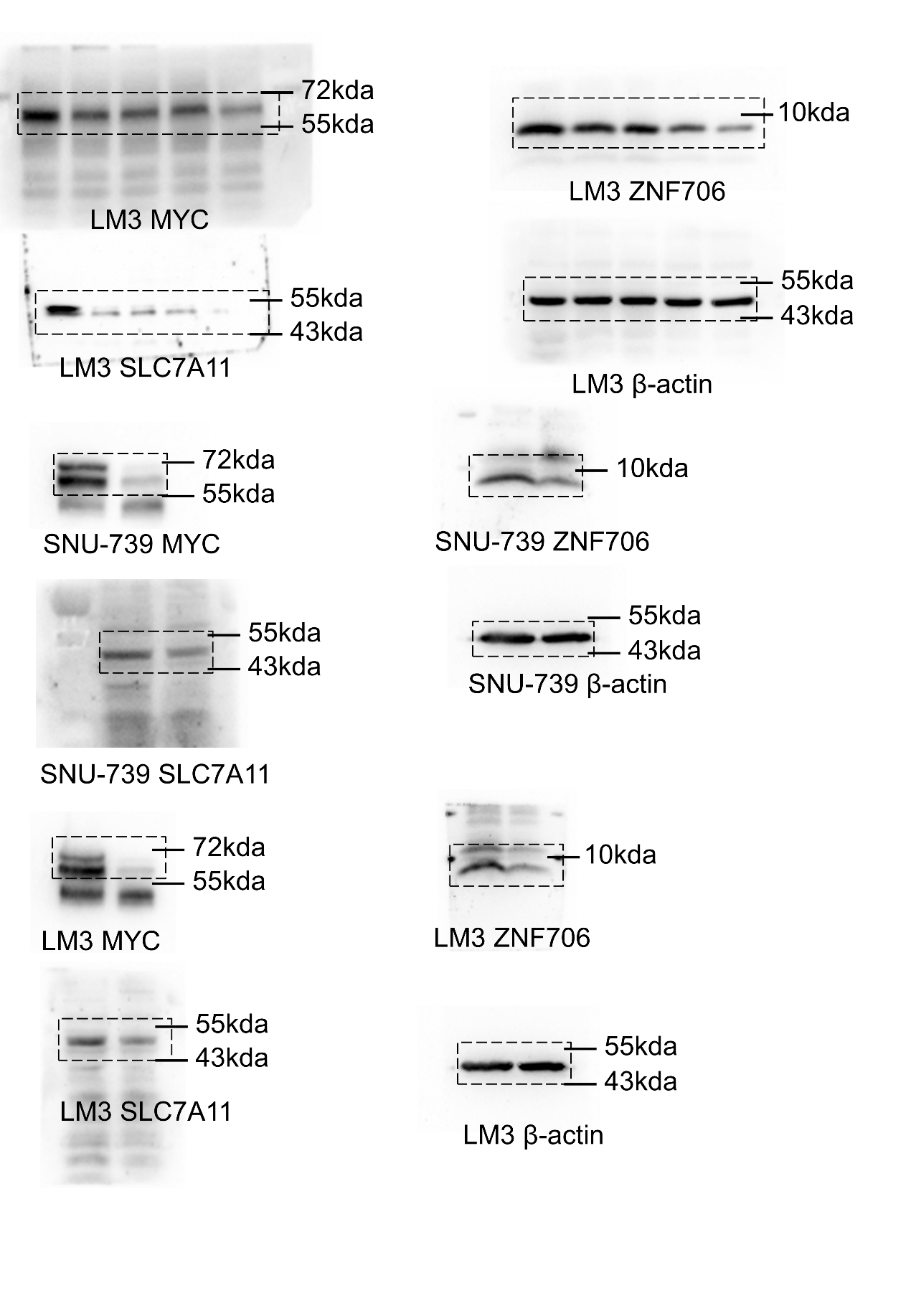

Supplement: Supplementary file 2 — Original Data File [file 41418_2024_1324_MOESM2_ESM.docx]
